# Modeling the effects of thin filament near-neighbor cooperative interactions in mammalian myocardium

**DOI:** 10.1085/jgp.202413582

**Published:** 2025-01-27

**Authors:** Tuan A. Phan, Daniel P. Fitzsimons

**Affiliations:** 1 Institute for Modeling Collaboration and Innovation, University of Idaho, Moscow, ID, USA; 2 Department of Animal, Veterinary, and Food Sciences, College of Agricultural and Life Sciences, University of Idaho, Moscow, ID, USA

## Abstract

The mechanisms underlying cooperative activation and inactivation of myocardial force extend from local, near-neighbor interactions involving troponin-tropomyosin regulatory units (RU) and crossbridges (XB) to more global interactions across the sarcomere. To better understand these mechanisms in the hearts of small and large mammals, we undertook a simplified mathematical approach to assess the contribution of three types of near-neighbor cooperative interactions, i.e., RU-induced, RU-activation (RU–RU), crossbridge-induced, crossbridge-binding (XB–XB), and XB-induced, RU-activation (XB–RU). We measured the Ca^2+^ and activation dependence of the rate constant of force redevelopment in murine- and porcine-permeabilized ventricular myocardium. Mathematical modeling of these three near-neighbor interactions yielded nonlinear expressions for the RU–RU and XB–RU rate coefficients (*k*_on_ and *k*_off_) and XB–XB rate coefficients describing the attachment of force-generating crossbridges (*f* and *f’*). The derivation of single cooperative coefficient parameters (u = RU–RU, w = XB–RU, and v = XB–XB) permitted an initial assessment of the strength of each near-neighbor interaction. The parameter sets describing the effects of discrete XB–XB or XB–RU interactions failed to adequately fit the in vitro contractility data in either murine or porcine myocardium. However, the Ca^2+^ dependence of *k*tr in murine and porcine ventricular myocardium was well fit by parameter sets incorporating the RU–RU cooperative interaction. Our results indicate that a significantly stronger RU–RU interaction is present in porcine ventricular myocardium compared with murine ventricular myocardium and that the relative strength of the near-neighbor RU–RU interaction contributes to species-specific myocardial contractile dynamics in small and large mammals.

## Teaser

We have created a mathematical model to provide a better understanding of the contribution of contractile and regulatory proteins to the regulation of contraction of mammalian cardiac muscle.

## Introduction

In the mammalian heart, each beat is characterized by a series of pressure and volume changes that underlie the ventricular filling (i.e., relaxation) and the subsequent ejection (i.e., contraction) of an appropriate stroke volume. Contraction is considered a positive cooperative process, in which the rapid development of pressure arises from the synergistic activation of the thin filament by calcium (Ca^2+^) and strong-binding myosin crossbridges (Razumova et al., 2000; Campbell et al., 2001; Lehrer and Geeves, 2014; Desai et al., 2015; Moore et al., 2016; Solis and Solaro, 2021). During relaxation, the dissociation of Ca^2+^ from troponin C (TnC) and the subsequent detachment of myosin crossbridges from actin results in the inactivation of the thin filament. This leads to an abrupt fall in pressure, which increases in ventricular compliance and augments ventricular filling. Thus, the sequential activation and inactivation of the myocardial thin filament due to cooperative mechanisms have profound implications for beat-to-beat ventricular systolic and diastolic function (Hanft et al., 2008; Solís and Solaro, 2021), particularly when considered in the context of a submaximal release of Ca^2+^ during a cardiac twitch (Bers, 2002). Evidence for the modulation of force development by cooperative processes is inferred from the observation of the nearly 10-fold variation in crossbridge kinetics as the [Ca^2+^] is increased from threshold to maximal levels in rodent myocardium (Stelzer et al., 2006a; Giles et al., 2019). The steep activation dependence of the kinetics of force development (*k*tr) can be explained, at least in part, by a conceptual model describing the cooperative effects associated with crossbridge binding and thin filament near-neighbor interactions (e.g., between adjacent regulatory units) that collectively act to slow crossbridge kinetics at low levels of Ca^2+^ activation (Campbell, 1997; Razumova et al., 2000; Campbell et al., 2001). While near-neighbor interactions can reasonably explain, at least in part, the cooperative development of force in mammalian myocardium, it should be emphasized that a number of mathematical models have been designed to investigate other potential determinants of thin filament activation. Each of these sophisticated models has examined the effects of various cooperative determinants, including end-to-end tropomyosin (Tm) interactions (Farman et al., 2010; Smith et al., 2003; Smith and Geeves, 2003), a crossbridge-mediated increase in the Ca^2+^-binding kinetics of TnC (Yadid et al., 2011; Kreutziger et al., 2011), and more recently, a role for thick filament activation (Caremani et al., 2022; Brunello and Fusi, 2024). For instance, in their elegant model, Smith and colleagues demonstrated that properties inherent in a flexible chain of actin-Tm-troponin provides a coherent basis for the cooperative binding of myosin to actin (Smith and Geeves, 2003). Of particular importance was their observation that the solution-based binding data could be fit without the overt need for incorporating near-neighbor cooperative factors. Although there is an assortment mathematical models, each utilizing a differing mathematical approach (e.g., inclusion or exclusion of parametric factors) and/or investigating a divergent array of cooperative determinants, an essential point has emerged. That is, these models collectively demonstrate that cooperativity is a fundamental property of mammalian myocardium, and its multifarious nature necessitates the continual investigation of its molecular and physical components.

It is well established that across mammalian species myocardial contractile kinetics are determined in part by the ventricular myosin heavy chain isoform (MyHC) expression. All mammalian species express both α- and β-MyHC isoforms in ventricular myocardium. However, the ratios of the two MyHC isoforms differ dramatically across species to account for the nearly 10-fold difference in beat frequency and contractile kinetics between small and large mammals. For example, with a resting heart rate of nearly 600 beats per minute, the murine ventricle primarily expresses the fast α-MyHC isoform, whereas large mammals (e.g., swine and human) predominately (i.e., ≥90%) express the comparatively slower β-MyHC isoform (Reiser et al., 2001; Sadayappan et al., 2009; Locher et al., 2011; Deacon et al., 2012; Walklate et al., 2016, 2021). Furthermore, myocardial twitch kinetics are also dictated by the responsiveness of the thin filament to the activating effects of Ca^2+^ and crossbridges, as mediated via local, near-neighbor (Razumova et al., 2000; Campbell et al., 2010; Kalda and Vendelin, 2020) and longer range interactions (Tanner et al., 2007, 2012; Moore et al., 2016; Dupuis et al., 2016). To examine whether molecular cooperativity underlies the species-dependent differences in contractile kinetics, we measured the activation dependence of the rate of force development in murine and porcine myocardium (Patel et al., 2023). We observed that the activation-dependent profile of *k*tr was significantly different between the two species. Although the rate of force redevelopment varied by a factor of three as the level of Ca^2+^ was raised from intermediate to maximal levels in porcine myocardium, near maximal *k*tr values were observed at very low levels of Ca^2+^ activation. Furthermore, in murine myocardium, the pCa_50_ for *k*tr was significantly right-shifted compared with the pCa50 for steady-state force. That is, increases in steady-state force occurred well before increases in the rate of force development were observed. In contrast, porcine ventricular myocardium was characterized by a tighter coupling between steady-state force and *k*tr, such that Ca^2+^-dependent increases in steady-state force were accompanied by simultaneous increases in the kinetics of force development (Patel et al., 2023). Therefore, as an initial step to better understand the cooperative mechanisms underlying myocardial contractile dynamics in the hearts of large and small mammals (Fig. S2), we undertook a simplified mathematical approach to quantify the contribution of thin filament near-neighbor cooperative interactions. This information is critical because the relative strength of near-neighbor interactions will collectively contribute to the extent of cooperative spread along the thin filament and subsequent force development. Our mathematical analysis focused on the three types of near-neighbor interactions: (1) an XB–XB interaction (defined by parameter *v*), in which the binding of a crossbridge cooperatively recruits the binding of neighboring crossbridges; (2) an XB–RU interaction (defined by parameter *w*), in which a strongly bound crossbridge cooperatively activates a neighboring RU; and (3) an RU–RU interaction (defined by parameter *u*), whereby an activated RU cooperatively activates a neighboring RU. Our model contains several unique adaptations from previously published models (Razumova et al., 2000; Campbell et al., 2010; Kalda and Vendelin, 2020). These include the further extension of the cooperative parameter *u* to define the effect of RU–RU cooperative interactions more clearly. First, we introduce the use of the cooperative coefficients *u*_1_ and *u*_2_ to describe the strength of neighboring RU–RU interactions and their impacts on the transitions between the blocked and closed states. Second, we propose the use of the cooperative coefficients *z*_1_ and *z*_2_ to delineate how neighboring RU–RU interactions impact the transitions between the closed and open states. Third, we derived nearest neighbor interaction factors, designated as $\alpha ,\bar{\alpha },\beta ,\bar{\beta }$, to measure the extent to which each of the near-neighbor RU–RU, XB–XB, and XB–RU cooperative interactions influence the transition between the blocked-to-closed and closed-to-open states of the thin filament. Collectively, our model demonstrates that the near-neighbor RU–RU interaction plays a principal role in the cooperative activation of force in both murine and porcine myocardium. However, species-specific differences in the relative strengths of the RU–RU, XB–XB, and XB–RU cooperative interactions underlie the differences in myocardial contractile dynamics in the hearts of small and large mammals.

## Materials and methods

### Model description

**Figure 1.**
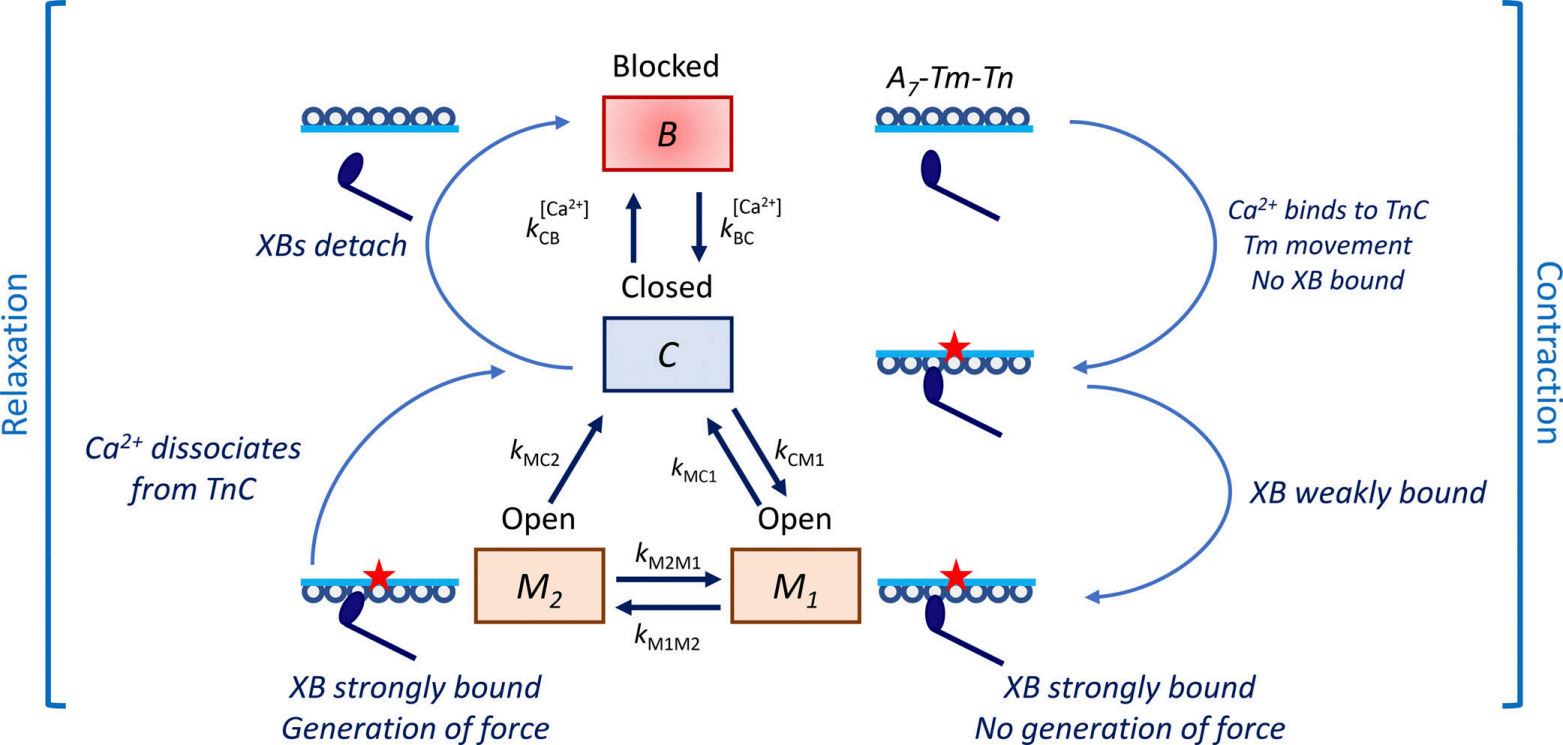
**Schematic of the model.** The model integrates three states of a single RU, consisting of blocked (B), closed (C), and open (M), and two states of an attached XB, including strongly bound pre-power stroke (M_1_) and strongly bound postpower stroke (M_2_), into a coupled four-state system. A thin filament RU is represented by a Tm bar associated with the seven-circle actin chain, with a myosin XB represented by the ellipse with a tail. A RU is blocked when the Tm bar is below the chain. A RU is in the closed state when Ca^2+^ binds to TnC (represented by a small red star) and the Tm bar is above the chain, with a XB weakly bound. A RU is in the open state when a XB is strongly bound. A XB may be detached (C) or attached (M_1_ and M_2_) to the thin filament. In isometric conditions, force generation occurs in the strongly bound postpower stroke state, M_2_.

Our model combines the processes of thin filament activation (three states) and crossbridge cycling (two states) into a coupled four-state system (Fig. 1) that is adapted from previous models (Razumova et al., 2000; Campbell et al., 2010). These collections of states represent an ensemble of myosin heads, associated actins, and regulatory proteins of the thick and thin filament. The blocked state *B* represents the blocked state of a RU along thin filament that prevents the formation of strongly bound XBs. The closed state *C* stands for the closed state of a RU with the nearest XB assumed to be in either a detached or weakly bound state. State *M*_1_ represents an open state of a RU in which nearest XB is strongly bound but is not generating force (i.e., contributes to the stiffness of the XB). The *M*_2_ state is considered an open state of a RU in which the nearest XB is strongly bound and generating force. A RU in the *M*_2_ state can return to the *C* state, where the XB head is detached or weakly bound. The *M*_2_-to-*C* state transition is unidirectional. The crossbridge cycle is assumed to consume one ATP. In contrast, the other state transitions are bidirectional and do not involve ATP hydrolysis.

The model formulation is based on the following assumptions:

(1) Ca^2+^ binding to TnC releases the inhibition of TnI on actin. The dissociation of Ca^2+^ from TnC reverses this process.
(2) Tm transitions from the blocked to the closed state only when TnI is dissociated from actin (i.e., Ca^2+^ binding to TnC induces the transition of Tm from blocked-to-closed state).
(3) Tm must be in the blocked state before TnI can rebind to actin and Ca^2+^ can dissociate from TnC.
(4) Strong binding of myosin places and holds Tm in the open state.
(5) Each RU regulates a single myosin-binding site.
(6) Ca^2+^ binding within a RU cannot directly expose binding sites in adjacent RUs.
(7) RU and XB interactions are considered along a single infinitely long thin filament (no end effects). We have not modeled interactions between multiple thick and thin filaments.
(8) Sarcomere length-dependent activation is not considered.
(9) States are assumed to be randomly distributed along the length of the myofilament (which is Bragg–Williams mean field approximation in statistical physics). This simplification helps avoid the requirement for probabilistic Monte–Carlo methods in computing solutions, which facilitates the use of an ordinary differential equation system, thereby reducing computation cost.
(10) The total number of actin-myosin binding sites is fixed.

### Mathematical model

Our model contains a series of four differential equations:

$$\dot{B}=k_{CB}C-k_{BC}B,$$ (1)

$$\dot{C}=k_{BC}B+k_{M_{2}C}M_{2}+k_{M_{1}C}M_{1}-\left(k_{CB}+k_{CM_{1}}\right)C,$$ (2)

$${\dot{M}}_{1}=k_{CM_{1}}C+k_{M_{2}M_{1}}M_{2}-\left(k_{M_{1}C}+k_{M_{1}M_{2}}\right)M_{1},$$ (3)

$${\dot{M}}_{2}=k_{M_{1}M_{2}}M_{1}-\left(k_{M_{2}C}+k_{M_{2}M_{1}}\right)M_{2}.$$ (4)

From assumption 10, in which the total number of actin-myosin–binding sites is fixed (*R*_*T*_), *B *+ *C *+ *M*_1_ + *M*_2_ = *R*_*T*_. Here, we denote *B* as the number of RUs that are in the blocked state and *C* as the number of RUs that are in the closed state along the thin filament. *M*_1_ and *M*_2_ are referred to as the number of RUs that are in the *M*_1_ and *M*_2_ state, respectively. A system of three differential equations (Eqs. 2, 3, and 4) is sufficient to describe the rate of change of states in our four–state model. The rate constants *k*_*BC*_ and *k*_*CB*_ describing the transitions between state *B* and state *C* depend on Ca^2+^ concentration as in the following:

$$k_{BC}=k_{BC}^{0}+\left[k_{BC}^{{\text{Ca}}^{2+}}-k_{BC}^{0}\right]\frac{{\text{Ca}}^{2+}}{{\text{Ca}}_{50}^{2+}+{\text{Ca}}^{2+}},$$ (5)

$$k_{CB}=k_{CB}^{0}+\left[k_{CB}^{{\text{Ca}}^{2+}}-k_{CB}^{0}\right]\frac{{\text{Ca}}^{2+}}{{\text{Ca}}_{50}^{2+}+{\text{Ca}}^{2+}},$$ (6)

in which ${\text{Ca}}_{50}^{2+}$ is the Ca^2+^ concentration of thin filament-binding sites at which the ratio in two above formulas equals 0.5. We only consider conditions of constant Ca^2+^ activation. By assumption 5, we also regard *B* as the number of XBs that are in the unbounded state and *C* as the number of XBs that are in the weakly bound state. *M*_1_ and *M*_2_ are also considered as the number of XBs that are in the strongly bound non-force–generating state and in the strongly bound force-generating state, respectively. Notice that, in some subsections below, we sometimes utilize *M* to indicate that an RU is in open state or a XB is in either *M*_1_ or *M*_2_ state.

The total crossbridge population is divided into two subpopulations of non-cycling (state *B*) and cycling crossbridges (i.e., states *C*, *M*_*1*_, and *M*_*2*_). State *M*_*2*_ is the unique state that can generate force during isometric conditions, and hence, isometric muscle force is proportional to the number of cycling crossbridges in the *M*_*2*_ state.

Each state and the combinations of states may be expressed as a fraction of *R*_*T*_:

$\lambda^{B}=\frac{B}{R_{T}}$—fraction of RUs that are blocked or fraction of XBs that are in the unbounded state.
$\lambda^{C}=\frac{C}{R_{T}}$—fraction of RUs that are closed or fraction of XBs that are in the weakly bound state.
$\lambda^{M}=\frac{M_{1}+M_{2}}{R_{T}}$—fraction of RUs that are open or fraction of XBs that are either in *M*_1_ or *M*_2_ state.
$\lambda^{M_{1}}=\frac{M_{1}}{R_{T}}$—fraction of RUs that are in *M*_1_ state or fraction of XBs that are in the strongly bound non-force–generating state.
$\lambda^{M_{2}}=\frac{M_{2}}{R_{T}}$—fraction of RUs that are in *M*_2_ state or fraction of XBs that are in the strongly bound force-generating state.

Due to assumption 9, the above fractions represent the probability that a given RU or XB is in any one state. For simplicity, from now on we assume that *R*_T_ = 1. Hence, $\lambda^{B}=B,\lambda^{C}=C,\lambda^{M_{1}}=M_{1}$ and $\lambda^{M_{2}}=M_{2}$.

Below, we briefly present how nearest neighbor RU–RU, XB–XB, and XB–RU interactions affects the transition rates *k*_*BC*_, *k*_*CB*_, $k_{CM_{1}}$ and $k_{M_{1}C}$ and the subsequent combination of all three nearest neighbor interactions into our thin filament model. For detailed presentation, we refer readers to Data S1.

#### RU–RU interactions

**Figure 2.**
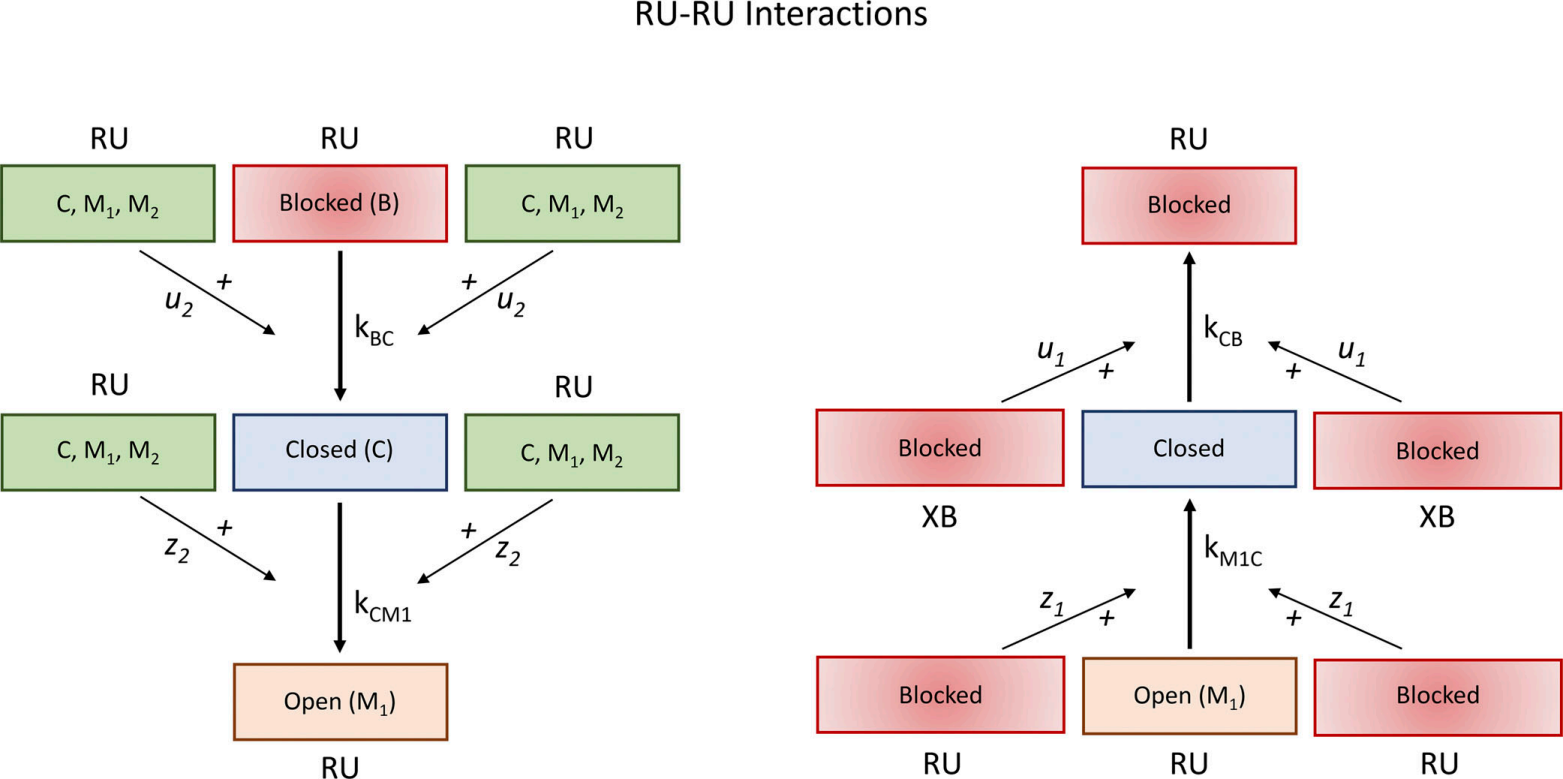
**Nearest neighbor cooperative interactions** **-** **Schematic of** **RU–RU interactions.** Left panel: The transition from a blocked to a closed state of an RU and the transition from a closed to an open state of an RU can be favored when its neighbor RUs are either in closed state or in open state. Right panel: The reversed transition from a closed to a blocked state of an RU and the reversed transition from an open to a closed state can be facilitated by its blocked neighbor RUs.

First, we formulate the effects of interactions between neighboring regulatory units on the blocked-to-closed (i.e., *k*_*BC*_ and *k*_*CB*_) and closed-to-open (i.e., $k_{CM_{1}}$ and $k_{M_{1}C}$) transitions (Fig. 2). We denote *u*_2_ as the cooperative coefficient that measures the strength of the effect of neighboring RU–RU interactions on the transition rate *k*_*BC*_ of a central RU when one of the neighboring RUs is in the *C*, *M*_1_, and *M*_2_ states. We denote *u*_1_ as the cooperative coefficient that measures the strength of the effect of neighboring RU–RU interactions on the transition rate *k*_*CB*_ of a central RU when one of the neighboring RUs is in the *B* state. We then let $k_{BC}^{\left(1,1\right)}$ be the reference blocked-to-closed transition *k*_*BC*_ and $k_{CB}^{\left(1,1\right)}$ be the reference closed-to-blocked transition *k*_*CB*_ under the condition where both neighboring RUs are in the closed state. The transition rates *k*_*BC*_ and *k*_*CB*_ are given by the following equations:

$$k_{BC}:=k_{BC}^{\left(u_{1},u_{2}\right)}=k_{BC}^{\left(1,1\right)}{\left[1-\lambda^{B}\left(1-u_{1}^{-1}\right)+\lambda^{M}\left(u_{2}-1\right)\right]}^{2},$$ (7)

$$k_{CB}:=k_{CB}^{\left(u_{1},u_{2}\right)}=k_{CB}^{\left(1,1\right)}{\left[1+\lambda^{B}\left(u_{1}-1\right)-\lambda^{M}\left(1-u_{2}^{-1}\right)\right]}^{2}.$$ (8)

The coefficient *u*_2_ is proportional to the activation energy needed to be overcome for the transition from the *B* state to the *C* state, and while the coefficient *u*_1_ is proportional to the activation energy needed to be overcome for the transition from the *C* state to the *B* state. Because the effects of Ca^2+^ on *k*_*BC*_ and *k*_*CB*_ are independent of the effects of neighbor interactions, $k_{BC}^{\left(1,1\right)}$ and $k_{CB}^{\left(1,1\right)}$ incorporate the Ca^2+^ effect as given by Eqs. 5 and 6.

Similarly, we let *z*_2_ be the cooperative coefficient that measures the strength of the effect of neighboring RU–RU interactions on the transition rate $k_{CM_{1}}$ of a central RU when one of the neighboring RUs is in one of the *C*, *M*_1_, and *M*_2_ states. We signify *z*_1_ as the cooperative coefficient that measures the strength of the effect of neighboring RU–RU interactions on the transition rate $k_{M_{1}C}$ of a central RU when one of the neighboring RUs is in the *B* state. We can then denote by $k_{CM_{1}}^{\left(1,1\right)}$ the reference closed-to-open transition $k_{CM_{1}}$ and $k_{M_{1}C}^{\left(1,1\right)}$ as the reference open-to-closed transition $k_{M_{1}C}$ under the condition where both neighboring RUs are in the closed state. The transition rates $k_{CM_{1}}$ and $k_{M_{1}C}$ can be described by the following equations:

$$k_{CM_{1}}:=k_{CM_{1}}^{\left(z_{1},z_{2}\right)}=k_{CM_{1}}^{\left(1,1\right)}{\left[1-\lambda^{B}\left(1-z_{1}^{-1}\right)+\lambda^{M}\left(z_{2}-1\right)\right]}^{2},$$ (9)

$$k_{M_{1}C}:=k_{M_{1}C}^{\left(z_{1},z_{2}\right)}=k_{M_{1}C}^{\left(1,1\right)}{\left[1+\lambda^{B}\left(z_{1}-1\right)-\lambda^{M}\left(1-z_{2}^{-1}\right)\right]}^{2}.$$ (10)

The coefficient *z*_2_ is proportional to the activation energy needed to be overcome for the transition from the *C* state to the *M*_1_ state and *z*_1_ from the *M*_1_ state to the *C* state.

#### XB–XB interactions

**Figure 3.**
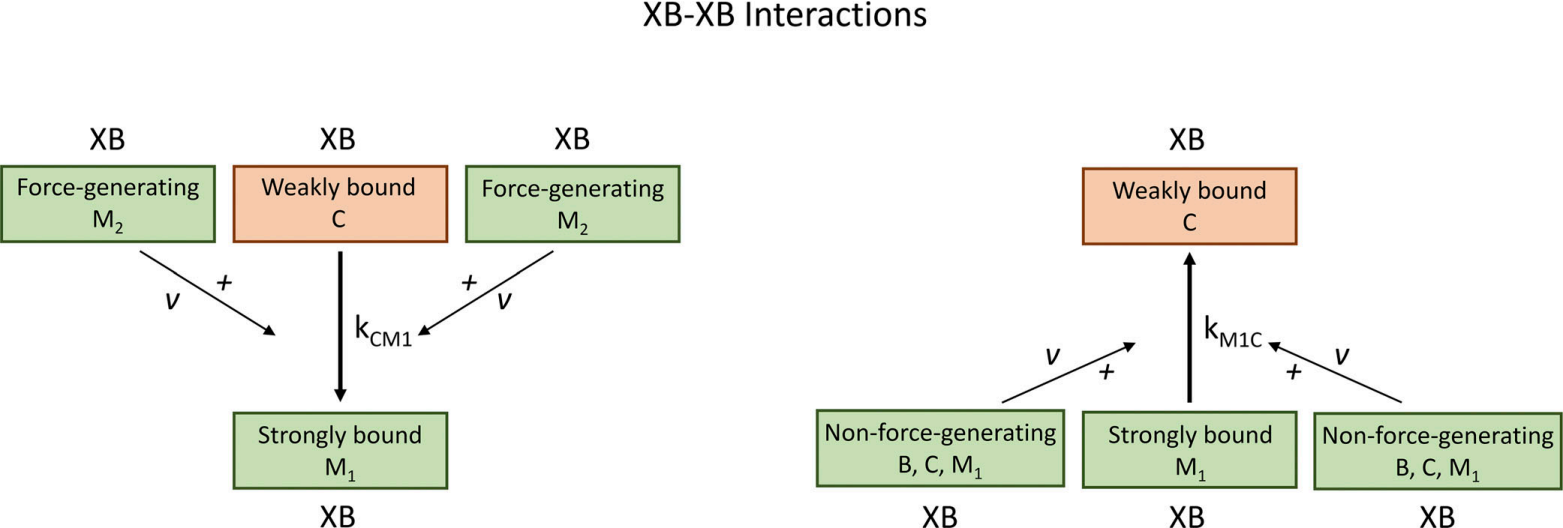
**Nearest neighbor cooperative interactions - Schematic of XB-XB interactions.** Left panel: A strongly bound force generating XB facilitates the transition from a weakly bound to a strongly bound state of a neighbor XB. Right panel: A non-force generating XB (B, C, M_1_) eases the reversed transition from a strongly bound to a weakly bound state of a neighbor XB.

Second, we consider that the force-generating state of an attached XB (i.e., *M*_2_) reduces the activation energy for the closed-to-open transition by increasing the rate coefficient $k_{CM_{1}}$ and decreasing the reverse rate coefficient $k_{M_{1}C}$ (Fig. 3). We use *v* to represent a cooperative parameter that measures the strength of neighboring XB–XB interactions on the transition rates $k_{CM_{1}}$ and $k_{M_{1}C}$. We denote $f_{CM_{1}}^{0}$ as the reference closed-to-open transition $k_{CM_{1}}$ under the condition where no neighboring XBs are in the force-generating state. We designate $f_{M_{1}C}^{0}$ as the reference closed-to-open transition $k_{CM_{1}}$ where no neighboring XBs are in the force-generating state. As a result, the transition rates $k_{CM_{1}}$ and $k_{M_{1}C}$ can be given by the following equations:

$$k_{CM_{1}}:=k_{CM_{1}}^{v}=f_{CM_{1}}^{0}{\left[1+\lambda^{M_{2}}\left(e^{v-1}-1\right)\right]}^{2},$$ (11)

$$k_{M_{1}C}:=k_{M_{1}C}^{v}=f_{M_{1}C}^{0}{\left[1+\lambda^{M_{2}}\left(e^{-v+1}-1\right)\right]}^{2}.$$ (12)

Note that the parameter *v* is closely related to the activation energy required for the transition between the closed and open states.

#### XB–RU interactions

**Figure 4.**
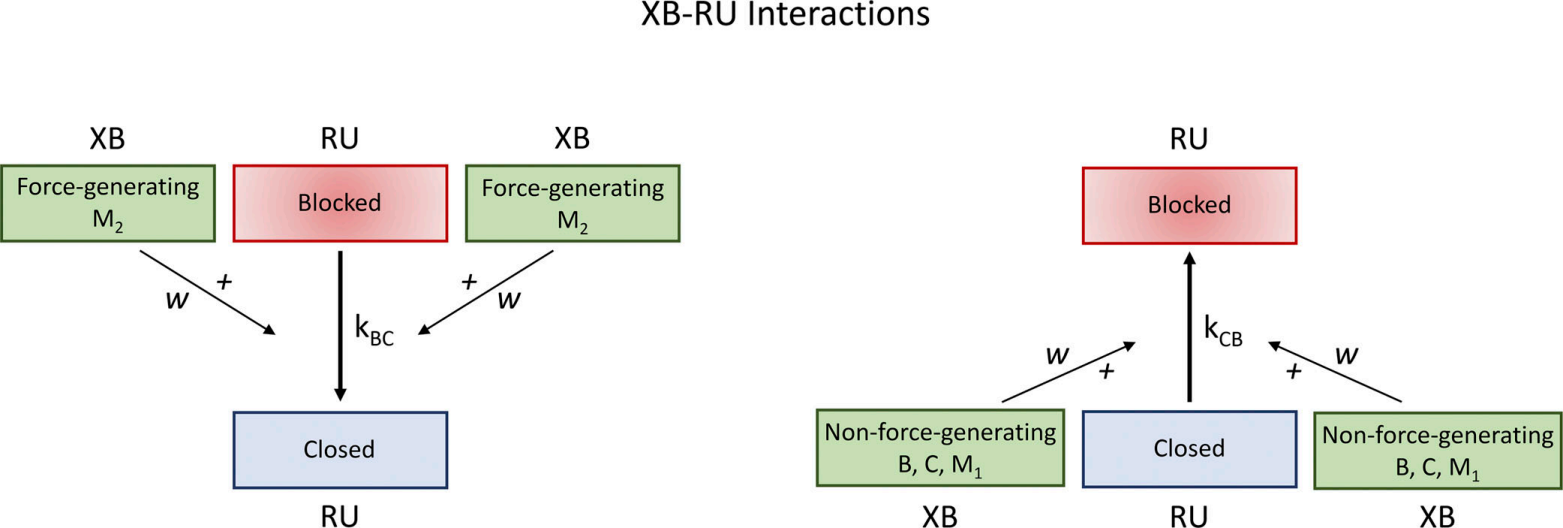
**Nearest neighbor cooperative interactions - Schematic of XB-RU interactions.** Left panel: A strongly bound force generating XB postively influences the transition of a near neighbor RU from the blocked to the closed state. Right panel: A non-force generating XB (B,C, M_1_) helps facilitate the reverse transition from a closed to a blocked state of a neighbor RU.

Third, we consider that the blocked-to-closed transition of RUs is favored by the force-generating state of XBs at neighboring actin-myosin attachment sites (Fig. 4). We allow that a force-generating XB at a neighboring site reduces the activation energy needed for the blocked-to-closed transition. Let *w* represent a cooperative parameter that measures the strength of neighboring XB–RU interactions on the transition rates *k*_*BC*_ and *k*_*CB*_. We indicate $f_{BC}^{0}$ as the reference blocked-to-closed transition *k*_*BC*_ and $f_{CB}^{0}$ as the reference closed-to-blocked transition *k*_*CB*_ under the condition where no neighboring XBs are in the force-generating state. Thus, the transition rates *k*_*BC*_ and *k*_*CB*_ can be delineated by the following equations:

$$k_{BC}:=k_{BC}^{w}=f_{BC}^{0}{\left[1+\lambda^{M_{2}}\left(e^{w-1}-1\right)\right]}^{2},$$ (13)

$$k_{CB}:=k_{CB}^{w}=f_{CB}^{0}{\left[1+\lambda^{M_{2}}\left(e^{-w+1}-1\right)\right]}^{2}.$$ (14)

Notice that the parameter *w* is closely related to the activation energy required for the transition between the blocked and closed states.

#### Ensemble effects of RU–RU, XB–XB, and XB–RU near-neighbor interactions

Finally, we can integrate the three nearest neighbor RU–RU, XB–XB, and XB–RU interactions into our thin filament model. We assume that the blocked-to-closed transitions and the closed-to-open transitions of an RU can be simultaneously impacted by the configurations of its two neighboring RUs and configurations of crossbridges at two neighboring actin-myosin attachment sites. In particular, we let 0 ≤ *α* ≤ 1 and 1−*α* be measures of the extent to which *k*_*BC*_ is affected by RU–RU and XB–RU interactions and $0\leq \bar{\alpha }\leq 1$ and $1-\bar{\alpha }$ be measures of the extent to which *k*_*CB*_ is affected by RU–RU and XB–RU interactions, respectively. In a similar fashion, we can denote by 0 ≤ *β* ≤ 1 and 1−*β* be measures of the extent to which $k_{CM_{1}}$ is affected by RU–RU and XB–XB interactions and $0\leq \bar{\beta }\leq 1$ and $1-\bar{\beta }$ be measures of the extent to which $k_{M_{1}C}$ is affected by RU–RU and XB–XB interactions, respectively. Then all the transition rates can be rewritten as

$$k_{BC}=f_{BC}^{0}\left\{\alpha {\left[1-\lambda^{B}\left(1-u_{1}^{-1}\right)+\lambda^{M}\left(u_{2}-1\right)\right]}^{2}+\left(1-\alpha \right){\left[1+\lambda^{M_{2}}\left(e^{w-1}-1\right)\right]}^{2}\right\},$$ (15)

$$k_{CB}=f_{CB}^{0}\left\{\bar{\alpha }{\left[1+\lambda^{B}\left(u_{1}-1\right)-\lambda^{M}\left(1-u_{2}^{-1}\right)\right]}^{2}+\left(1-\bar{\alpha }\right){\left[1+\lambda^{M_{2}}\left(e^{-w+1}-1\right)\right]}^{2}\right\},$$ (16)

$$k_{CM_{1}}=f_{CM_{1}}^{0}\left\{\beta {\left[1-\lambda^{B}\left(1-z_{1}^{-1}\right)+\lambda^{M}\left(z_{2}-1\right)\right]}^{2}+\left(1-\beta \right){\left[1+\lambda^{M_{2}}\left(e^{v-1}-1\right)\right]}^{2}\right\},$$ (17)

$$k_{M_{1}C}=f_{M_{1}C}^{0}\left\{\bar{\beta }{\left[1+\lambda^{B}\left(z_{1}-1\right)-\lambda^{M}\left(1-z_{2}^{-1}\right)\right]}^{2}+\left(1-\bar{\beta }\right){\left[1+\lambda^{M_{2}}\left(e^{-v+1}-1\right)\right]}^{2}\right\}.$$ (18)

For convenience, we designate the parameters $\left(\alpha ,\bar{\alpha },\beta ,\bar{\beta }\right)$ as nearest neighbor interaction factors. Model state variables and equations (Table S2) and transitions rate coefficients, near-neighbor cooperative coefficients, and nearest neighbor interaction factors (Table S3) are summarized in the Data S1.

#### In silico modeling Ca^2+^-activated force and the activation dependence of *k*tr

**Figure S1.**
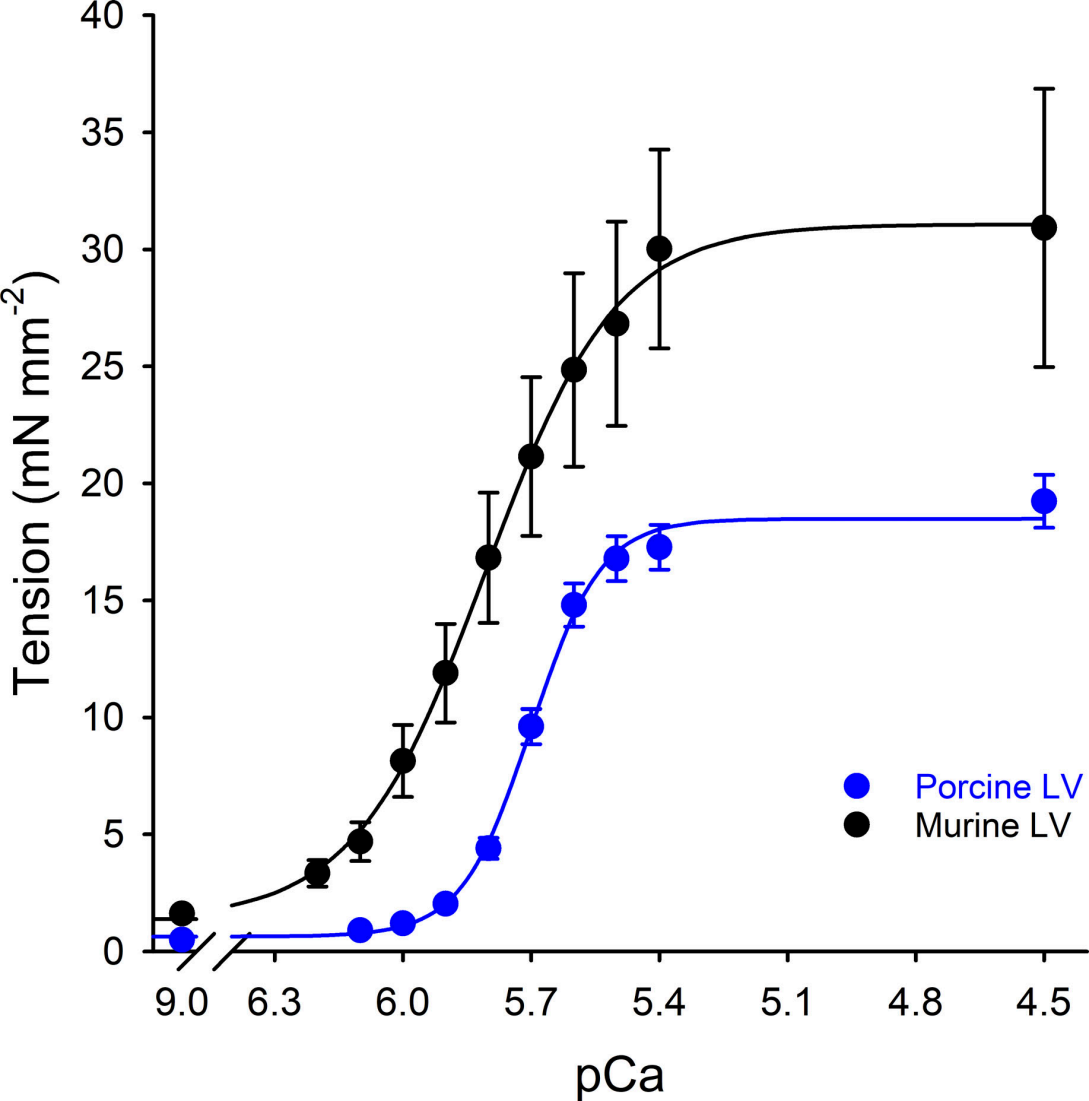
**Steady-state tension development in mammalian-permeabilized myocardium.** Ca^2+^-activated tension–pCa relationship was measured in murine- (black circle) and porcine- (blue circle) permeabilized ventricular myocardium. All values represent means, and error bars represent ± SEM.

**Figure S2.**
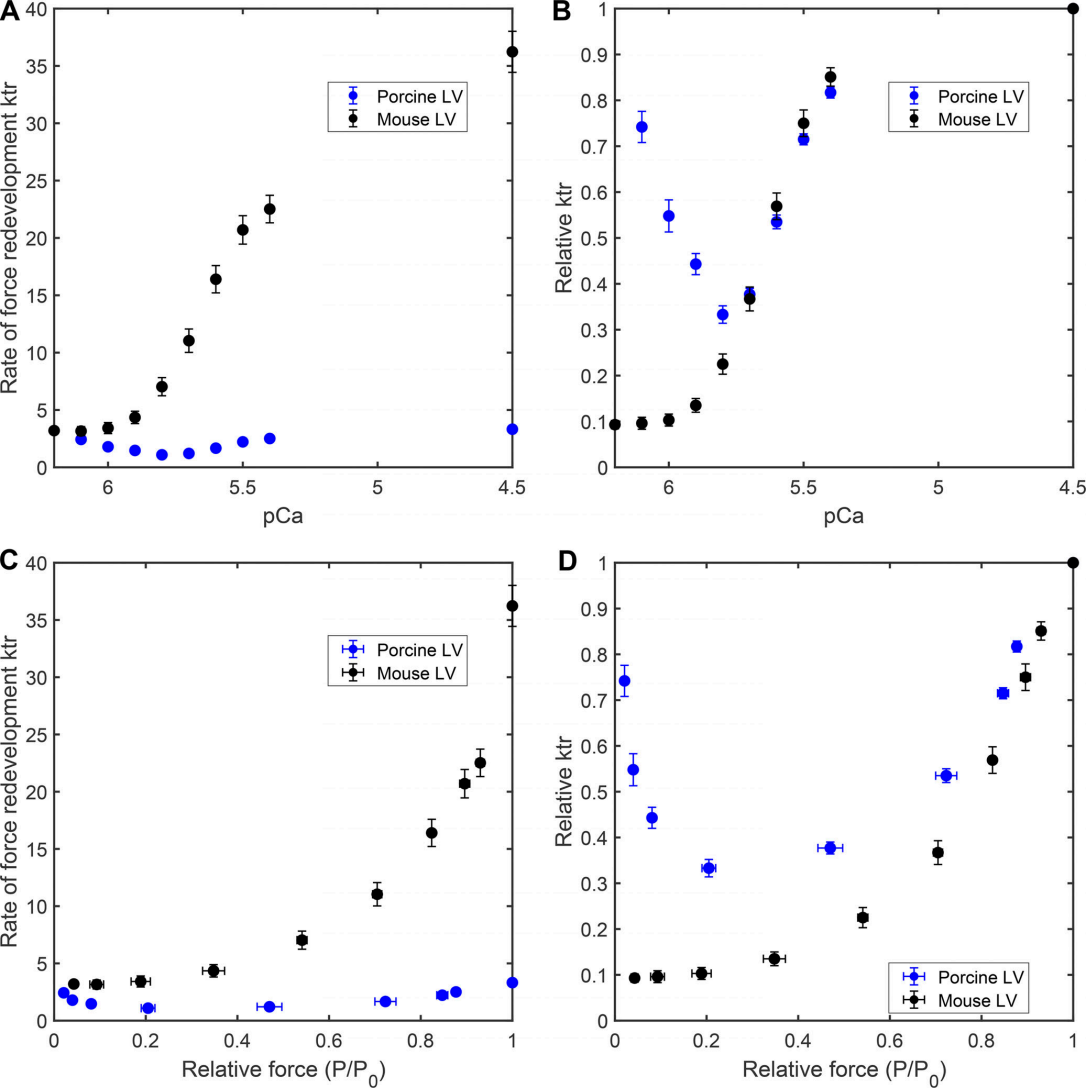
**Ca** ^**2+**^ **and activation dependence of the rate of force redevelopment in mammalian-skinned myocardium.** The rate constant of force redevelopment (*k*tr) following a rapid release/restretch maneuver was measured in permeabilized myocardial preparations isolated de novo from murine ventricular myocardium (black-filled circles) and porcine ventricular myocardium (blue-filled circles, from Patel et al. [2023]). Data points represent the means, and the error bars are the SEM (Table S1). **(A and B)** The Ca^2+^ dependence of the rate constant of force redevelopment (A) and relative *k*tr (B). **(C and D)** The activation dependence of the rate constant of force redevelopment (C) and relative *k*tr (D).

In vitro contractility measurements of relative force (*P/P*_o_), *k*tr and relative *k*tr (i.e., *k*tr_submax_/*k*tr_max_) values collected from murine- and porcine-permeabilized left ventricular myocardium are summarized in Figs. S1 and S2. The in vitro–derived mechanical measurements were modeled using the eight discrete parameter sets. In silico force–pCa relationships were generated by running the model for each ${\text{Ca}}^{2+}/{\text{Ca}}_{50}^{2+}$ value until steady-state force is reached (i.e., the last value of *M*_2_). If we denote *P* as the submaximal Ca^2+^-activated force, *P*_0_ as the maximal Ca^2+^-activated force, $pCa_{50}=-\log_{10}\left[Ca_{50}^{2+}\right]$ as the Ca^2+^ sensitivity of force development (which is the Ca^2+^ concentration required for half maximal activation), and *pCa* = −log_10_[*Ca*^2+^] as the Ca^2+^ concentration for activating force *P*, then the force–pCa relationship can be represented via the Hill equation:

$$\frac{P}{P_{0}}=\frac{1}{1+{\left(\frac{\left[Ca_{50}^{2+}\right]}{\left[Ca^{2+}\right]}\right)}^{n}}=\frac{1}{1+10^{n\left(pCa-pCa_{50}\right)}}$$

where *n* is Hill coefficient (giving the estimate of the minimum number of binding sites involved in the regulation of force). The Ca^2+^ and activation dependence of the rate of force redevelopment was modeled in silico by initially determining the steady-state force (i.e., *F*_*ss*_) for a given ${\text{Ca}}^{2+}/{\text{Ca}}_{50}^{2+}$. We then modeled the *k*tr slack-restretch maneuver (Patel et al., 2023) as a transient lowering of the duty cycle, which is defined by the ratio:

$$\delta =\frac{f_{CM_{1}}^{0}}{f_{CM_{1}}^{0}+f_{M_{1}C}^{0}}.$$

This was performed by initially setting *δ* = 10^−8^ and then running the model with this new duty cycle until the steady-state force is reached. The last value of *M*_2_ gives the nonzero residual force *F*_resid_. Finally, by using this steady-state solution for initial conditions including *F*_resid_, the duty cycle is restored to its normal value, and the model is evolved in time until the steady state is achieved, giving the steady-state force *F*_*ss*_. Thus:

$$ktr=-\frac{\ln\left(1/2\right)}{t_{1/2}}$$

where *t*_1/2_ is the time course from the restretch required for force to reach *F*_resid_ + 0.5(*F*_*ss*_ − *F*_resid_) from *F*_resid_.

**Figure S3.**
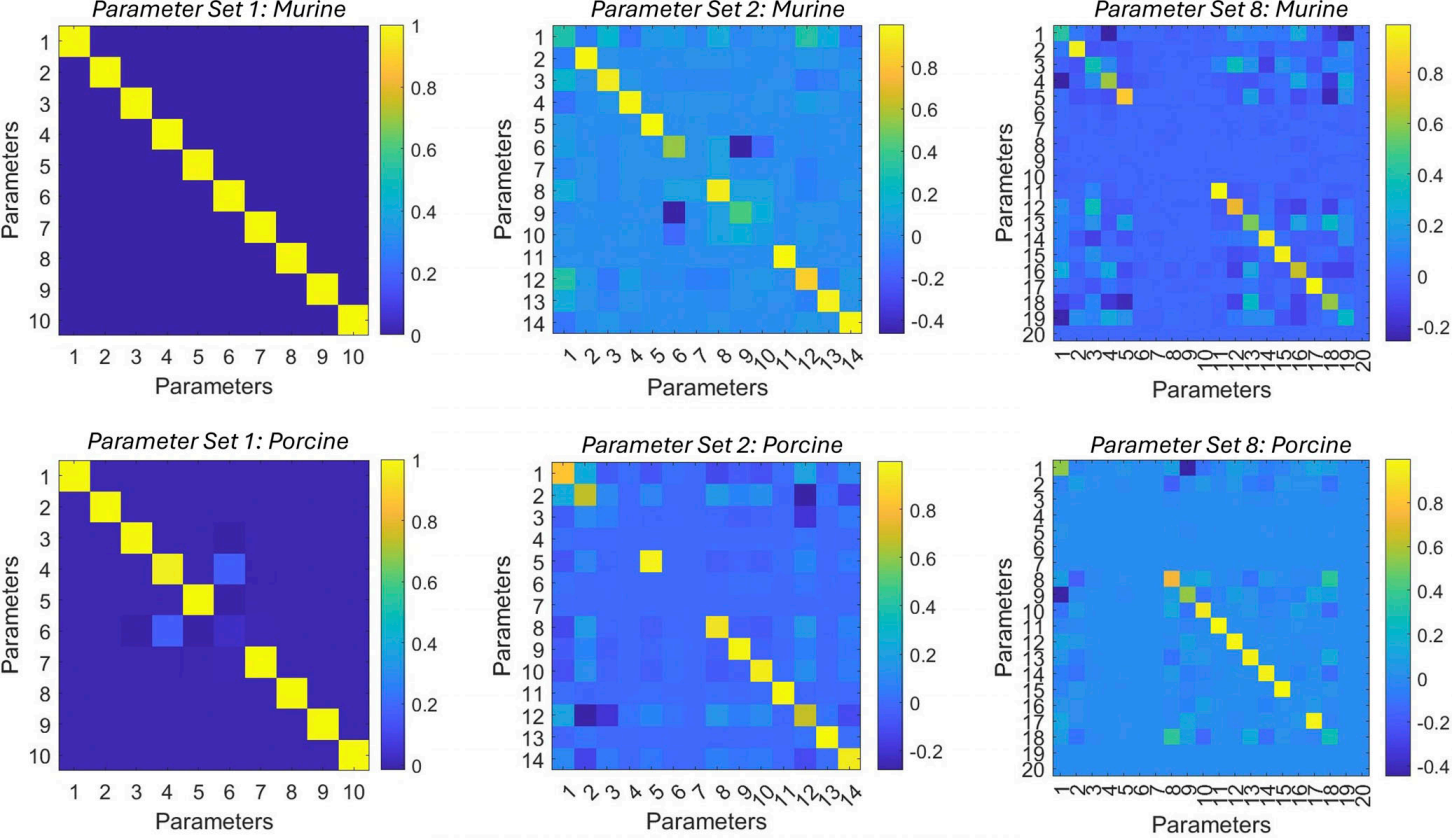
**Model resolution matrices generated by fitted results for modeling the Ca** ^**2+**^ **dependence of the rate of force redevelopment in mammalian myocardium.** Numerical identification of rate coefficients, cooperative coefficients (*u*_*1*_, *u*_*2*_, *z*_*1*_, *z*_*2*_, *v*, *and w*) and nearest neighbor interaction factors (α, α, β, and β) are summarized in Table S5. Upper panels represent the model resolution matrices for murine myocardium for parameter set 1 (left), parameter set 2 (middle), and parameter set 8 (right). Lower panels represent the model resolution matrices for porcine myocardium for parameter set 1 (left), parameter set 2 (middle), and parameter set 8 (right).

A detailed description of the mathematical model and materials and methods used in the present study can be found in the Fig. S3; and Tables S4 and S5.

### Online supplemental material

**Figure S4.**
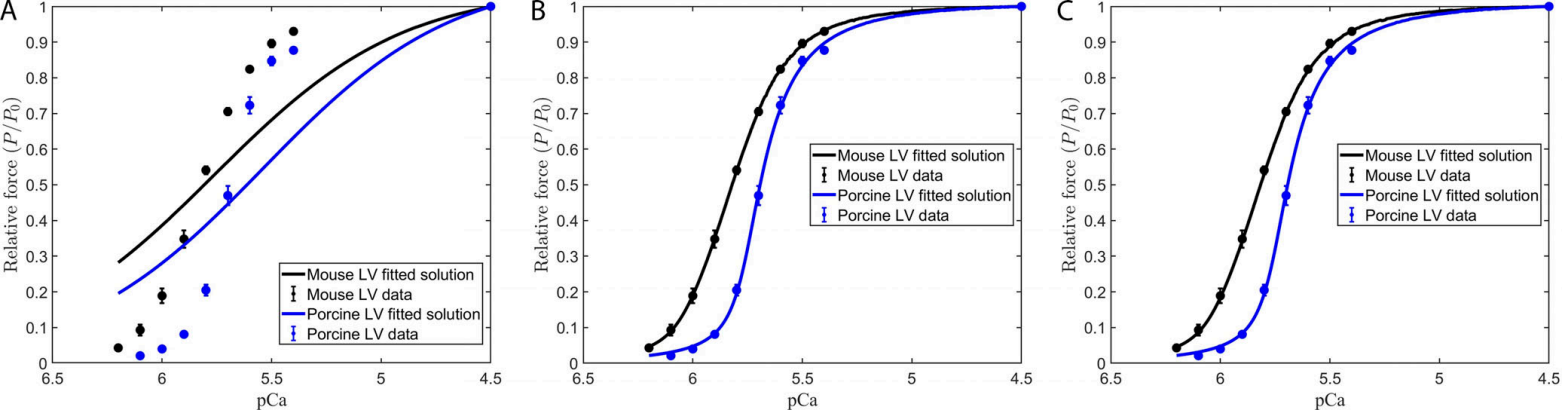
**Modeling the effect of near-neighbor cooperative interactions on the force–pCa relationship in mammalian myocardium.** Ca^2+^-activated relative force (P/P_0_) was measured in murine- (black circles) and porcine- (blue circles) permeabilized ventricular myocardium. All values represent means, and error bars represent ± SEM (from Table S1). **(A–C)**Eqs. 2, 3, and 4 were used in conjunction with parameter set 1 (A), parameter set 2 (B), and parameter set 8 (C) to fit to the force–pCa data. Fitted parameters are shown in Table S4.

Fig. S1 shows the steady-state tension development in mammalian-permeabilized myocardium. Fig. S2 shows the Ca^2+^- and activation-dependence of the rate of force redevelopment in mammalian-skinned myocardium. Fig. S3 shows the model resolution matrices generated by fitted results for modeling the Ca^2+^ dependence of the rate of force redevelopment in mammalian myocardium. Fig. S4 shows modeling the effect of near-neighbor cooperative interactions on the force–pCa relationship in mammalian myocardium. Table S1 shows the summary of steady-state mechanical data in murine- and porcine-permeabilized ventricular myocardium. Table S2 shows the summary of state variables and model equations. Table S3 shows the summary of rate coefficients, cooperative coefficients, and nearest neighbor interaction factors. Table S4 shows the fitted parameters for modeling force–pCa relationships in murine- and porcine-permeabilized ventricular myocardium. Table S5 shows the parameter identification for model resolution matrices. Data S1 shows the steady-state contractile measurements.

## Results

The kinetics of myofilament activation and crossbridge cycling were described by a four-state model, utilizing a system of three ordinary differential equations (Eqs. 2, 3, and 4) under a conservative constraint that the total number of actin-myosin sites along the whole thin filament is fixed. The effect of Ca^2+^ to activate RUs is represented by the dependence of the rate constants *k*_*BC*_ and *k*_*CB*_ on Ca^2+^ given by Eqs. 5 and 6. Without any nearest neighbor interaction, $k_{BC}=k_{BC}^{\left(1,1\right)}=f_{BC}^{0}$, $k_{CB}=k_{CB}^{\left(1,1\right)}=f_{CB}^{0}$, $k_{CM_{1}}=k_{CM_{1}}^{\left(1,1\right)}=f_{CM_{1}}^{0}$, and $k_{M_{1}C}=k_{M_{1}C}^{\left(1,1\right)}=f_{M_{1}C}^{0}$.

When all nearest neighbor interactions RU–RU, XB–XB, XB–RU are taken into consideration, *k*_*BC*_, *k*_*CB*_, $k_{CM_{1}}$, and $k_{M_{1}C}$ become nonlinear expressions given by Eqs. 15, 16, 17, and 18. The system of three ordinary differential equations (Eqs. 2, 3, and 4) constitutes a nonlinear third-order dynamical model (Rice et al., 2008; Land et al., 2017) in the state variables (*C*, *M*_1_, and *M*_2_). All inputs are held constant and dynamic behavior is the result of responses to nonsteady-state initial conditions. Model output predicted a steady-state force that, under the isometric conditions, is proportional to *M*_2_. In total, our model contains 20 parameters: 9 reference values for the rate coefficients are as follow:

$$\left(k_{BC}^{0},k_{BC}^{{\text{Ca}}^{2+}},k_{CB}^{0},k_{CB}^{{\text{Ca}}^{2+}},f_{CM_{1}}^{0},f_{M_{1}C}^{0},k_{M_{1}M_{2}},k_{M_{2}M_{1}},k_{M_{2}C}\right),$$

the ${\text{Ca}}^{2+}/{\text{Ca}}_{50}^{2+}$ ratio, 6 cooperative parameters (*u*_1_, *u*_2_, *z*_1_, *z*_2_, *v*, and *w*) measuring the strength of RU–RU (i.e., *u*_1_, *u*_2_, *z*_1_, and *z*_2_), XB–XB (i.e., *v*), and XB–RU (i.e., *w*) near-neighbor interactions, and four nearest neighbor interaction factors $\left(\alpha ,\bar{\alpha },\beta ,\text{and}\;\bar{\beta }\right)$ measuring the extent to which each near-neighbor interaction effects the forward and reverse blocked-to-closed and closed-to-open transitions. We integrated the model (Eqs. 2, 3, and 4) using the fourth-order Runge–Kutta method to predict steady-state force during varying levels of constant Ca^2+^ activation (represented by changing the pCa = −log[Ca^2+^]) and the time course of force redevelopment (*k*tr) starting from zero-force initial conditions.

### Creation of parameter sets

We designed eight discrete parameter sets using assorted values of nearest neighbor interaction factors and cooperative coefficients $\left(\alpha ,\bar{\alpha },\beta ,\bar{\beta },u_{1},u_{2},z_{1},z_{2},v,\text{and}\;w\right)$ to quantify the unitary, tandem, and ensemble effects of the RU–RU, XB–XB, and XB–RU cooperative interactions (Table 1).

**Table 1.** Model parameter sets: Cooperative coefficients and nearest neighbor interaction factors

Combinations	Set	*u*_1_, *u*_2_	*z*_1_, *z*_2_	*v*	*w*	α	α	β	β
No cooperative interaction	1	1, 1	1, 1	1	1	[0, 1]	[0, 1]	[0, 1]	[0, 1]
Unitary interaction RU–RU XB–XB XB–RU	2 3 4	*u*_1_ > 1 or *u*_2_ > 1 1, 1 1, 1	*z*_1_ > 1 or *z*_2_ > 1 1, 1 1, 1	1 >1 1	1 1 >1	1 [0, 1] 0	1 [0, 1] 0	1 0 [0, 1]	1 0 [0, 1]
Tandem interactions RU–RU and XB–XB XB–XB and XB–RU RU–RU and XB–RU	5 6 7	*u*_1_ > 1 or *u*_2_ > 1 1, 1 *u*_1_ ≥ 1 or *u*_2_ ≥ 1	*z*_1_ ≥ 1 or *z*_2_ ≥ 1 1, 1 *z*_1_ > 1 or *z*_2_ > 1	>1 >1 1	1 >1 >1	1 0 [0, 1)	1 0 [0, 1)	[0, 1) 0 1	[0, 1) 0 1
Ensemble interactions RU–RU, XB–XB, and XB–RU	8	*u*_1_ > 1 or *u*_2_ > 1	*z*_1_ > 1 or *z*_2_ > 1	>1	>1	[0, 1]	[0, 1]	[0, 1]	[0, 1]

Eight parameter sets were designed to vary the strength (*u*_*1*_, *u*_*2*_, *z*_*1*_, *z*_*2*_, *v*, and *w*) and extent (α, α, β, and β) of near-neighbor cooperative interactions on the rate constants *k*_BC_, *k*_CB_, *k*_CM1_, and *k*_M1C_.

#### Parameter set 1: No near-neighbor cooperative interactions

In this scenario, we set *u*_1_ = *u*_2_ = *z*_1_ = *z*_2_ = *v* = *w* = 1 to exclude all near-neighbor cooperative interactions. Regardless of the values of $\left(\alpha ,\bar{\alpha },\beta ,\text{and}\;\bar{\beta }\right)$, the transition rates $k_{BC},k_{CB},{\;k}_{CM_{1}}$, and $k_{M_{1}C}$ will be described by

$$k_{BC}=k_{BC}^{\left(1,1\right)}=k_{BC}^{0}+\left[k_{BC}^{Ca^{2+}}-k_{BC}^{0}\right]\frac{\left[Ca^{2+}\right]}{\left[Ca_{50}^{2+}\right]+\left[Ca^{2+}\right]},$$

$$k_{CB}=k_{CB}^{\left(1,1\right)}=k_{CB}^{0}+\left[k_{CB}^{Ca^{2+}}-k_{CB}^{0}\right]\frac{\left[Ca^{2+}\right]}{\left[Ca_{50}^{2+}\right]+\left[Ca^{2+}\right]},$$

$$k_{CM_{1}}=f_{CM_{1}}^{0},k_{M_{1}C}=f_{M_{1}C}^{0}.$$

#### Parameter set 2: Unitary effect of the RU–RU interaction

In this scenario, we set either *u*_1_ > 1 or *u*_2_ > 1 and either *z*_1_ > 1 or *z*_2_ > 1 to examine the RU–RU interaction, with *v* = 1 and *w* = 1 to eliminate the XB–XB and XB–RU cooperative interactions. By setting all nearest neighbor interaction factors to 1 (i.e., $\left[\alpha ,\bar{\alpha },\beta ,\text{and}\;\bar{\beta }\right]=\left[1,1,1,\text{and}\;1\right]$), the transition rates $k_{BC},k_{CB},\;k_{CM_{1}}$, and $k_{M_{1}C}$ will be described by

$$k_{BC}=k_{BC}^{\left(1,1\right)}{\left[1-\left(1-C-M_{1}-M_{2}\right)\left(1-u_{1}^{-1}\right)+\left(M_{1}+M_{2}\right)\left(u_{2}-1\right)\right]}^{2},$$

$$k_{BC}^{\left(1,1\right)}=k_{BC}^{0}+\left[k_{BC}^{Ca^{2+}}-k_{BC}^{0}\right]\frac{\left[Ca^{2+}\right]}{\left[Ca_{50}^{2+}\right]+\left[Ca^{2+}\right]},$$

$$k_{CB}=k_{CB}^{\left(1,1\right)}{\left[1+\left(1-C-M_{1}-M_{2}\right)\left(u_{1}-1\right)-\left(M_{1}+M_{2}\right)\left(1-u_{2}^{-1}\right)\right]}^{2},$$

$$k_{CB}^{\left(1,1\right)}=k_{CB}^{0}+\left[k_{CB}^{Ca^{2+}}-k_{CB}^{0}\right]\frac{\left[Ca^{2+}\right]}{\left[Ca_{50}^{2+}\right]+\left[Ca^{2+}\right]},$$

$$k_{CM_{1}}=f_{CM_{1}}^{0}{\left[1-\left(1-C-M_{1}-M_{2}\right)\left(1-z_{1}^{-1}\right)+\left(M_{1}+M_{2}\right)\left(z_{2}-1\right)\right]}^{2},$$

$$k_{M_{1}C}=f_{M_{1}C}^{0}{\left[1+\left(1-C-M_{1}-M_{2}\right)\left(z_{1}-1\right)-\left(M_{1}+M_{2}\right)\left(1-z_{2}^{-1}\right)\right]}^{2}.$$

#### Parameter set 3: Unitary effect of the XB–XB interaction

In this scenario, we set *v* > 1 to examine the XB–XB interaction and *u*_1_ = *u*_2_ = 1, *z*_1_ = *z*_2_ = 1, and *w* = 1 to eliminate the RU–RU and XB–RU cooperative interactions. By setting $\beta =\bar{\beta }=0$ and allowing the $\left(\alpha ,\bar{\alpha }\right)$ values to vary between 0 and 1, the transition rates $k_{BC},k_{CB},{\;k}_{CM_{1}}$, and $k_{M_{1}C}$ will be described by

$$k_{BC}=k_{BC}^{0}+\left[k_{BC}^{Ca^{2+}}-k_{BC}^{0}\right]\frac{\left[Ca^{2+}\right]}{\left[Ca_{50}^{2+}\right]+\left[Ca^{2+}\right]},$$

$$k_{CB}=k_{CB}^{0}+\left[k_{CB}^{Ca^{2+}}-k_{CB}^{0}\right]\frac{\left[Ca^{2+}\right]}{\left[Ca_{50}^{2+}\right]+\left[Ca^{2+}\right]},$$

$$k_{CM_{1}}=f_{CM_{1}}^{0}{\left[1+M_{2}\left(e^{v-1}-1\right)\right]}^{2},$$

$$k_{M_{1}C}=f_{M_{1}C}^{0}{\left[1+M_{2}\left(e^{-v+1}-1\right)\right]}^{2}.$$

#### Parameter set 4: Unitary effect of the XB–RU interaction

In this scenario, we set *w* > 1 to examine the XB–RU interaction and *u*_1_ = *u*_2_ = 1, *z*_1_ = *z*_2_ = 1, and *v* = 1 to eliminate the RU–RU and XB–XB cooperative interactions. By setting $\alpha =\bar{\alpha }=0$ and allowing the $\left(\beta ,\bar{\beta }\right)$ values to vary between 0 and 1, the transition rates $k_{BC},k_{CB},{\;k}_{CM_{1}}$, and $k_{M_{1}C}$ in the model will be described by

$$k_{BC}=f_{BC}^{0}{\left[1+M_{2}\left(e^{w-1}-1\right)\right]}^{2},$$

$$f_{BC}^{0}=k_{BC}^{0}+\left[k_{BC}^{Ca^{2+}}-k_{BC}^{0}\right]\frac{\left[Ca^{2+}\right]}{\left[Ca_{50}^{2+}\right]+\left[Ca^{2+}\right]},$$

$$k_{CB}=f_{CB}^{0}{\left[1+M_{2}\left(e^{-w+1}-1\right)\right]}^{2},$$

$$f_{CB}^{0}=k_{CB}^{0}+\left[k_{CB}^{Ca^{2+}}-k_{CB}^{0}\right]\frac{\left[Ca^{2+}\right]}{\left[Ca_{50}^{2+}\right]+\left[Ca^{2+}\right]},$$

$$k_{CM_{1}}=f_{CM_{1}}^{0},k_{M_{1}C}=f_{M_{1}C}^{0}.$$

#### Parameter set 5: The tandem effects of RU–RU and XB–XB interactions

In this scenario, we set either *u*_1_ > 1 or *u*_2_ > 1, and either *z*_1_ ≥ 1 or *z*_2_ ≥ 1, and *v* > 1 to study the combined effects of the RU–RU and XB–XB interactions. We set *w* = 1 to eliminate the XB–RU cooperative interaction. By setting the values of $\left(\alpha ,\bar{\alpha }\right)=\left(1,1\right)$, 0 ≤ *β* < 1, and $0\leq \bar{\beta }<1$, the transition rates $k_{BC},k_{CB},{\;k}_{CM_{1}}$, and $k_{M_{1}C}$ will be described by

$$k_{BC}=k_{BC}^{\left(1,1\right)}{\left[1-\left(1-C-M_{1}-M_{2}\right)\left(1-u_{1}^{-1}\right)+\left(M_{1}+M_{2}\right)\left(u_{2}-1\right)\right]}^{2},$$

$$k_{BC}^{\left(1,1\right)}=k_{BC}^{0}+\left[k_{BC}^{Ca^{2+}}-k_{BC}^{0}\right]\frac{\left[Ca^{2+}\right]}{\left[Ca_{50}^{2+}\right]+\left[Ca^{2+}\right]},$$

$$k_{CB}=k_{CB}^{\left(1,1\right)}{\left[1+\left(1-C-M_{1}-M_{2}\right)\left(u_{1}-1\right)-\left(M_{1}+M_{2}\right)\left(1-u_{2}^{-1}\right)\right]}^{2},$$

$$k_{CB}^{\left(1,1\right)}=k_{CB}^{0}+\left[k_{CB}^{Ca^{2+}}-k_{CB}^{0}\right]\frac{\left[Ca^{2+}\right]}{\left[Ca_{50}^{2+}\right]+\left[Ca^{2+}\right]},$$

$$k_{CM_{1}}=f_{CM_{1}}^{0}\{\beta {\left[1-\left(1-C-M_{1}-M_{2}\right)\left(1-z_{1}^{-1}\right)+\left(M_{1}+M_{2}\right)\left(z_{2}-1\right)\right]}^{2}+\left(1-\beta \right){\left[1+M_{2}\left(e^{v-1}-1\right)\right]}^{2}\},$$

$$k_{M_{1}C}=f_{M_{1}C}^{0}\{\bar{\beta }{\left[1+\left(1-C-M_{1}-M_{2}\right)\left(z_{1}-1\right)-\left(M_{1}+M_{2}\right)\left(1-z_{2}^{-1}\right)\right]}^{2}+\left(1-\bar{\beta }\right){\left[1+M_{2}\left(e^{-v+1}-1\right)\right]}^{2}\}.$$

#### Parameter set 6: The tandem effects of XB–XB and XB–RU interactions

In this scenario, we set *v* > 1 and *w* > 1 to study the collective effects of the XB–XB and XB–RU interactions. We set *u*_1_ = *u*_2_ = 1 and *z*_1_ = *z*_2_ = 1 to eliminate the RU–RU cooperative interaction. By setting all nearest neighbor interaction values to 0 (i.e., $\left[\alpha ,\bar{\alpha },\beta ,\text{and}\;\bar{\beta }\right]=\left[0,0,0,\text{and}\;0\right]$), the transition rates $k_{BC},k_{CB},{\;k}_{CM_{1}}$, and $k_{M_{1}C}$ will be described by

$$k_{BC}=f_{BC}^{0}{\left[1+M_{2}\left(e^{w-1}-1\right)\right]}^{2},$$

$$f_{BC}^{0}=k_{BC}^{0}+\left[k_{BC}^{Ca^{2+}}-k_{BC}^{0}\right]\frac{\left[Ca^{2+}\right]}{\left[Ca_{50}^{2+}\right]+\left[Ca^{2+}\right]},$$

$$k_{CB}=f_{CB}^{0}{\left[1+M_{2}\left(e^{-w+1}-1\right)\right]}^{2},$$

$$f_{CB}^{0}=k_{CB}^{0}+\left[k_{CB}^{Ca^{2+}}-k_{CB}^{0}\right]\frac{\left[Ca^{2+}\right]}{\left[Ca_{50}^{2+}\right]+\left[Ca^{2+}\right]},$$

$$k_{CM_{1}}=f_{CM_{1}}^{0}{\left[1+M_{2}\left(e^{v-1}-1\right)\right]}^{2},$$

$$k_{M_{1}C}=f_{M_{1}C}^{0}{\left[1+M_{2}\left(e^{-v+1}-1\right)\right]}^{2}.$$

#### Parameter set 7: The tandem effects of RU–RU and XB–RU interactions

In this scenario, we set either *u*_1_ ≥ 1 or *u*_2_ ≥ 1, either *z*_1_ > 1 or *z*_2_ > 1, and *w* > 1 to examine the collective effects of the RU–RU and XB–RU interactions. We set *v* = 1 to eliminate the XB–XB cooperative interaction. By setting 0 ≤ *α* < 1, $0\leq \bar{\alpha }<1$, and $\left(\beta ,\bar{\beta }\right)=\left(1,1\right)$, the transition rates $k_{BC},k_{CB},\;k_{CM_{1}}$, and $k_{M_{1}C}$ will be described by

$$k_{BC}=f_{BC}^{0}\{\alpha {\left[1-\left(1-C-M_{1}-M_{2}\right)\left(1-u_{1}^{-1}\right)+\left(M_{1}+M_{2}\right)\left(u_{2}-1\right)\right]}^{2}+\left(1-\alpha \right){\left[1+M_{2}\left(e^{w-1}-1\right)\right]}^{2}\},$$

$$f_{BC}^{0}=k_{BC}^{0}+\left[k_{BC}^{Ca^{2+}}-k_{BC}^{0}\right]\frac{\left[Ca^{2+}\right]}{\left[Ca_{50}^{2+}\right]+\left[Ca^{2+}\right]},$$

$$k_{CB}=f_{CB}^{0}\{\bar{\alpha }{\left[1+\left(1-C-M_{1}-M_{2}\right)\left(u_{1}-1\right)-\left(M_{1}+M_{2}\right)\left(1-u_{2}^{-1}\right)\right]}^{2}+\left(1-\bar{\alpha }\right){\left[1+M_{2}\left(e^{-w+1}-1\right)\right]}^{2}\},$$

$$f_{CB}^{0}=k_{CB}^{0}+\left[k_{CB}^{Ca^{2+}}-k_{CB}^{0}\right]\frac{\left[Ca^{2+}\right]}{\left[Ca_{50}^{2+}\right]+\left[Ca^{2+}\right]},$$

$$k_{CM_{1}}=f_{CM_{1}}^{0}{\left[1-\left(1-C-M_{1}-M_{2}\right)\left(1-z_{1}^{-1}\right)+\left(M_{1}+M_{2}\right)\left(z_{2}-1\right)\right]}^{2},$$

$$k_{M_{1}C}=f_{M_{1}C}^{0}{\left[1+\left(1-C-M_{1}-M_{2}\right)\left(z_{1}-1\right)-\left(M_{1}+M_{2}\right)\left(1-z_{2}^{-1}\right)\right]}^{2}.$$

#### Parameter set 8: The ensemble effects of RU–RU, XB–XB, and XB–RU interactions

In this scenario, we set either *u*_1_ > 1 or *u*_2_ > 1, either *z*_1_ > 1 or *z*_2_ > 1, *v* > 1, and *w* > 1 to probe the ensemble effects of the RU–RU, XB–XB, and XB–RU cooperative interactions. By setting the nearest neighbor interaction factors as 0 < *α* < 1, $0<\bar{\alpha }<1$, 0< *β* < 1, and $0<\bar{\beta }<1$, the transition rates $k_{BC},k_{CB},\;k_{CM_{1}}$, and $k_{M_{1}C}$ will be described by

$$k_{BC}=f_{BC}^{0}\{\alpha {\left[1-\left(1-C-M_{1}-M_{2}\right)\left(1-u_{1}^{-1}\right)+\left(M_{1}+M_{2}\right)\left(u_{2}-1\right)\right]}^{2}+\left(1-\alpha \right){\left[1+M_{2}\left(e^{w-1}-1\right)\right]}^{2}\},$$

$$f_{BC}^{0}=k_{BC}^{0}+\left[k_{BC}^{Ca^{2+}}-k_{BC}^{0}\right]\frac{\left[Ca^{2+}\right]}{\left[Ca_{50}^{2+}\right]+\left[Ca^{2+}\right]},$$

$$k_{CB}=f_{CB}^{0}\{\bar{\alpha }{\left[1+\left(1-C-M_{1}-M_{2}\right)\left(u_{1}-1\right)-\left(M_{1}+M_{2}\right)\left(1-u_{2}^{-1}\right)\right]}^{2}+\left(1-\bar{\alpha }\right){\left[1+M_{2}\left(e^{-w+1}-1\right)\right]}^{2}\},$$

$$f_{CB}^{0}=k_{CB}^{0}+\left[k_{CB}^{Ca^{2+}}-k_{CB}^{0}\right]\frac{\left[Ca^{2+}\right]}{\left[Ca_{50}^{2+}\right]+\left[Ca^{2+}\right]},$$

$$k_{CM_{1}}=f_{CM_{1}}^{0}\{\beta {\left[1-\left(1-C-M_{1}-M_{2}\right)\left(1-z_{1}^{-1}\right)+\left(M_{1}+M_{2}\right)\left(z_{2}-1\right)\right]}^{2}\;+\left(1-\beta \right){\left[1+M_{2}\left(e^{v-1}-1\right)\right]}^{2}\},$$

$$k_{M_{1}C}=f_{M_{1}C}^{0}\{\bar{\beta }{\left[1+\left(1-C-M_{1}-M_{2}\right)\left(z_{1}-1\right)-\left(M_{1}+M_{2}\right)\left(1-z_{2}^{-1}\right)\right]}^{2}+\left(1-\bar{\beta }\right){\left[1+M_{2}\left(e^{-v+1}-1\right)\right]}^{2}\}.$$

### Modeling in vitro contractility data

**Figure 5.**
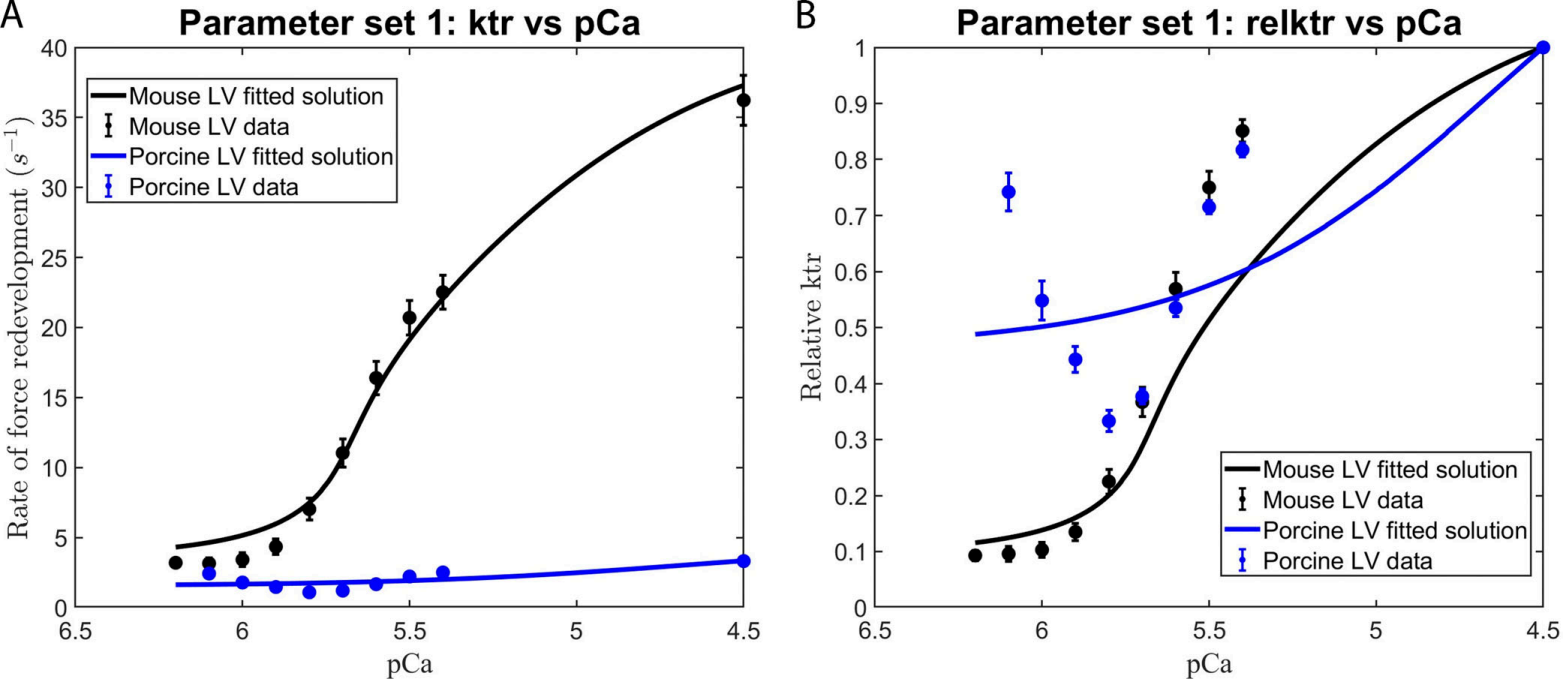
**Modeling the effect of RU–RU, XB–XB, and XB–RU cooperative interactions on the Ca** ^**2+**^ **dependence of the rate of force redevelopment in mammalian myocardium. (A and B)** Eqs. 2, 3, and 4 were used in conjunction with parameter set 1 (Table 2) to fit to the contractile data derived from the (A) *k*tr versus pCa and (B) relative ktr–pCa relationships. The rate constant of force redevelopment was measured in murine- and porcine-permeabilized ventricular myocardium (from Fig. S2). The data points (filled circles) represent the means, and the error bars are the SEM (from Table S1).

**Figure 6.**
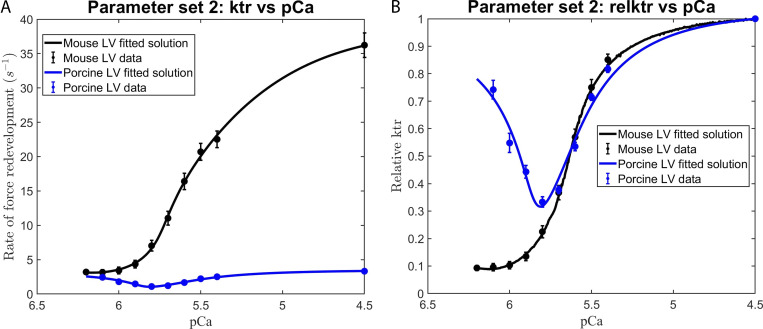
**Modeling the effects of RU-RU, XB-XB, and XB-RU cooperative interactions on the Ca** ^**2+**^ **dependence of the rate of force redevelopment in mammalian myocardium.** **(A and B)** Eqs. 2, 3, and 4 were used in conjunction with parameter set 2 (Table 2) to fit to the contractile data derived from the (A) *ktr* versus pCa and (B) relative *ktr* versus pCa relationships. The rate of constant of force redevelopment was measured in murine and porcine permeabilized ventricular myocardium (from Fig. S2). The data points (filled circles) represent the means and the error bars are the SEM (from Table S1).

**Figure 7.**
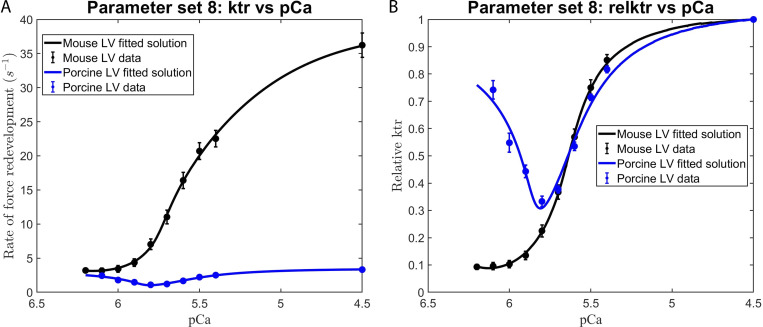
**Modeling the effects of RU-RU, XB-XB, and XB-RU cooperative interactions on the Ca^2+^ dependence of the rate of force redevelopment in mammalian myocardium.** **(A and B)** Eqs. 2, 3, and 4 were used in conjunction with parameter set 8 (Table 2) to fit to the contractile data derived from the (A) ktr versus pCa and (B) relative ktr versus pCa relationships. The rate constant of force redevelopment was measured in murine and porcine permeabilized ventricular myocardium (from Fig. S2). The data points (filled circles) represent the means and the error bars are the SEM (from Table S1).

We consistently observed that the use of those parameter sets that excluded the effects of the RU–RU cooperative interaction failed to adequately fit our in vitro contractility data. This observation is illustrated by the failure of parameter set 1 (i.e., the elimination of RU–RU, XB–XB, and XB–RU cooperative interactions) to adequately fit the force–pCa (Fig. S4 A) and *k*tr–pCa (Fig. 5) relationships in murine and porcine myocardium. In a similar manner, parameter set 3 (i.e., unitary effects of XB–XB cooperative interactions), parameter set 4 (i.e., unitary effects of XB–RU cooperative interactions), and parameter set 6 (i.e., the tandem effects of XB–XB and XB–RU cooperative interactions) failed to fit the force–pCa and ktr–pCa relationships (data not shown). In contrast, our in vitro contractility data obtained in murine- and porcine-permeabilized myocardium was well fit using parameter sets that included the RU–RU cooperative interaction, i.e., the unitary effects of RU–RU interaction (set 2), the tandem effects of RU–RU and XB–XB (set 5) and RU–RU and XB–RU interactions (set 7), and the ensemble effects of RU–RU, XB–XB, and RU–XB interactions (set 8). For simplicity, here we present the modeling results using parameter sets 2 and 8. To examine the unitary effect of the RU–RU cooperative interaction (i.e., parameter set 2), we fit our in vitro contractility data by allowing the RU–RU cooperative coefficients describing the blocked-closed transition (*u*_1_ and *u*_2_) and the closed-open transition (*z*_1_ and *z*_2_) to become more cooperative (i.e., attain values >1) and eliminated the cooperativity associated with near-neighbor XB–XB (i.e., *v = 1*) and XB–RU (i.e., *w = 1*) interactions. Further, we fixed the nearest neighbor interaction factors (i.e., $\alpha =\bar{\alpha }=\beta =\bar{\beta }=1$) to ensure that the RU–RU cooperative interaction was the sole component effecting the blocked-closed and closed-open transitions. The fitted parameters derived from the unitary RU–RU cooperative interaction are summarized in Table 2, and the in silico–derived *k*tr–pCa and rel *k*tr–pCa relationships are illustrated in Fig. 6. To provide a more physiologic framework for thin filament near-neighbor communication, we examined the ensemble effects of the RU–RU, XB–XB, and XB–RU cooperative interactions. To do so, we fit the in vitro contractility data using parameter set 8 in which the accompanying RU–RU, XB–XB, and XB–RU cooperative coefficients (i.e., *u*_1_, *u*_2_, *z*_1_, *z*_2_, *v*, and *w*) were permitted to attain values >1 (i.e., introducing cooperativity into these near-neighbor interactions). To examine the extent to which the RU–RU and XB–RU interactions effected the blocked-closed transition and the RU–RU and XB–XB interactions effected the closed-open transition, we let the nearest neighbor interaction factors vary between 0 and 1 (i.e., 0 < *α* < 1, $0<\bar{\alpha }<1$, 0 < *β* < 1, and $0<\bar{\beta }<1$). The fitted parameters derived from the ensemble effects of the RU–RU, XB–XB, and XB–RU interactions are summarized in Table 2, and the in silico–derived *k*tr–pCa and rel *k*tr–pCa relationships are illustrated in Fig. 7. The use of parameter set 2 and parameter set 8 produced remarkably similar fits of the in vitro contractility data. Both parameter sets produced large values for the cooperative coefficient *u*_2_ in murine and porcine myocardium (i.e., 2.2659 versus 17.0820; Table 2). A summary of the fraction of crossbridges in the non-cycling and cycling populations for parameter sets 1, 2, and 8 are presented in Table 3. Collectively, these results demonstrate that the interaction between near-neighbor RUs is the predominant cooperative mechanism for the spread of thin filament activation in mammalian myocardium as previously predicted (Campbell et al., 2010; Kalda and Vendelin, 2020). Furthermore, the larger fitted value of *u*_2_ in porcine myocardium indicates a relatively stronger RU–RU cooperative coupling exists within the porcine thin filament.

**Table 2.** Fitted parameters for modeling *k*tr–pCa relationships in murine and porcine permeabilized ventricular myocardium

	Murine parameter sets			Porcine parameter sets		
Parameter	Set 1	Set 2	Set 8	Set 1	Set 2	Set 8
pCa_50_	5.63	5.63	5.64	5.65	5.65	5.65
*k* ^0^_BC_	0.0166	0.0167	0.0	0.0	0.0	24.3511
*k* ^Ca2+^_BC_	0.4370	1.9370	1.9453	208.4857	243.0357	243.5360
*k* ^0^_CB_	2.3633	2.3633	2.3617	5,907.6526	1,694.7526	1,592.7530
*k* ^Ca2+^_CB_	0.3979	0.3979	0.3939	246.3005	0.0	117.5183
*f* ^0^_CM1_	72.6072	43.0072	42.9689	103.8109	91.0080	90.9918
*f* ^0^_M1C_	44.2949	50.2949	50.2944	157.6488	691.6738	691.7920
*k*_M1M2_	50.6703	39.5703	42.5863	14.6009	3.2005	2.6762
*k*_M2M1_	53.5794	47.3794	44.7155	1.6748	2.5001	2.5561
*k*_M2C_	55.7214	47.2714	47.8151	0.0367	0.7366	0.6945
*u*_1_	1.0	1.0	1.0054	1.0	1.0	1.1055
*u*_2_	1.0	2.2208	2.2659	1.0	15.098	17.0820
*z*_1_	1.0	1.0	1.0047	1.0	1.0	1.0001
*z*_2_	1.0	1.0	1.0118	1.0	1.1346	1.0
*V*	1.0	1.0	1.0067	1.0	1.0	1.0374
*W*	1.0	1.0	1.0463	1.0	1.0	1.0
Α	1.0	1.0	0.9996	1.0	1.0	1.0
Α	1.0	1.0	0.8897	1.0	1.0	0.8296
Β	1.0	1.0	0.9466	1.0	1.0	1.0
Β	1.0	1.0	0.1376	1.0	1.0	1.0
RMSE	3.7580	1.0501	1.1072	1.3499	0.2800	0.2696

Fitted parameters for *k*tr–pCa relationships in murine- and porcine-permeabilized myocardium using parameter set 1 (no near-neighbor cooperative interactions), parameter set 2 (describing the unitary effect of the RU–RU cooperative interaction), and parameter set 8 (describing the ensemble effects of RU–RU, XB–XB, and XB–RU cooperative interactions). RMSE, root mean squared error.

**Table 3.** Fitted parameters for modeling *k*tr–pCa relationships in murine- and porcine-permeabilized ventricular myocardium

	Murine parameter sets			Porcine parameter sets		
Parameter	Set 1	Set 2	Set 8	Set 1	Set 2	Set 8
K	0.0070	0.0298	7.70e-06	7.98e-08	8.80e-06	0.3312
N	1.6392	0.8551	0.8654	0.6585	0.1473	0.1315
B	0.3719	0.0362	0.0367	0.3050	0.0137	0.0339
C	0.2497	0.5159	0.5129	0.0957	0.7630	0.7794
M_1_	0.2584	0.3163	0.3085	0.0629	0.1123	0.1024
M_2_	0.1199	0.1316	0.1419	0.5365	0.1110	0.0843
λ_cyc_	0.6281	0.9638	0.9633	0.6950	0.9863	0.9661
λ_cyc_^M2^	0.1909	0.1365	0.1473	0.7719	0.1126	0.0873
*k*tr	37.2481	36.1691	36.1584	3.3395	3.3489	3.3452

Summary of computational results for the model fit of *k*tr–pCa relationship in murine and porcine myocardium (Table S1). The model is run with parameter sets 1, 2, and 8 with fitted rate constants taken from Table 2. The values of B, C, M_1_, and M_2_ are recorded when the solution of the ODE system Eqs. 2, 3, and 4 reach their equilibrium state at maximal activation. K, the activation factor $\left(K=k_{\text{BC}}/k_{\text{CB}}\right)$; N, the crossbridge recruitment factor $\left(N=k_{\text{CM}1}/k_{M1C}\right)$; λ_cyc_, the fraction of crossbridges participating in crossbridge cycling $\left(\lambda_{\text{cyc}}=C+M_{1}+M_{2}\right)$; and λ_cyc_^M2^, the fraction of cycling crossbridges that generate force $\left(\lambda_{\text{cyc}}^{M2}=M_{2}/\lambda_{\text{cyc}}\right)$ is computed based on these values. Finally, *k*tr is computed according to the slack-restretch maneuver described by Patel et al. (2023).

## Discussion

The regulation of contraction in mammalian cardiac muscle is a multivariable process triggered by the initial binding of Ca^2+^ to TnC, which in turn induces a switch-like effect on thin filament regulatory proteins to permit crossbridge binding to actin and the subsequent development of force (Solis and Solaro, 2021). It is important to stress that Ca^2+^-activated force is not a simple linear function of the myoplasmic [Ca^2+^]. Rather, the effect of Ca^2+^ binding to TnC is amplified by positive cooperativity in myosin crossbridge binding to actin, in which the initial binding of Ca^2+^ to TnC increases the likelihood of subsequent crossbridge binding to actin (Moss et al., 2004, 2015). These effects are mediated via local interactions along a segment of the thin filament within and between troponin-Tm regulatory units (Moore et al., 2016; Solís and Solaro, 2021). As a result, once a myosin crossbridge binds, the activation profile expands away from the central Ca^2+^-bound TnC, permitting the cooperative recruitment of additional crossbridge binding within the activated RU and among neighboring RUs (Moss et al., 2015). However, the relative strength of near-neighbor interactions (i.e., the degree of cooperativity between near-neighbors) will affect, at least in part, the extent of cooperative spread along the thin filament due to initial Ca^2+^ and crossbridge binding (Razumova et al., 2000; Campbell et al., 2010; Kalda and Vendelin, 2020). In murine ventricular myocardium, the rate constant of force redevelopment (*k*tr) exhibits a steep activation dependence, increasing nearly 10-fold across varying levels of Ca^2+^ activation (Stelzer et al., 2006a; Giles et al., 2019). In contrast, the activation dependence of *k*tr in porcine ventricular myocardium is significantly reduced, as manifested by the near-maximal rates of force redevelopment at low levels of Ca^2+^ activation. A critical question is what molecular mechanism(s) can account, at least in part, for the differing activation-dependent profiles of *k*tr in the hearts of small and large mammals?

At all levels of submaximal [Ca^2+^], porcine myocardium exhibited significantly faster relative rates of force development than murine myocardium, with the greatest difference in crossbridge kinetics observed at very low [Ca^2+]^. One possible way to explain the markedly divergent *k*tr values between porcine and murine myocardium at very low levels of Ca^2+^ activation is the binding of Ca^2+^ to a few discrete regions of the thin filament and the isolation of active RUs with Ca^2+^ bound from adjacent near-neighbor RUs having no Ca^2+^ bound (i.e., RUs in “blocked” state). The first condition may arise if the binding of Ca^2+^ to the thin filament is not uniform along the thin filament. This would result in the nonuniform (i.e., stochastic) activation of the thin filament (Risi et al., 2021), which would limit the number of strongly bound crossbridges and account for the small forces developed in both species. The second condition may result from species-dependent differences in the activation energy required to cooperatively recruit near-neighbor RUs. In porcine myocardium, the relatively higher activation energy required to cooperatively recruit near-neighbor RUs would effectively isolate and perhaps even eliminate near-neighbor RU communication at very low [Ca^2+]^. As a result, the small number of activated RUs in the porcine thin filament would have very few or no adjacent near-neighbor RUs with either Ca^2+^ or a crossbridge bound, thereby making the cooperative recruitment of crossbridges into the adjacent near-neighbor RUs less likely. If the length of the functional (i.e., cooperative) unit is essentially the length of a structural unit spanning seven actin monomers (Gillis et al., 2007), these effects would appreciably increase *k*tr.

At intermediate [Ca^2+]^, relative *k*tr values measured in porcine myocardium were less than that observed at low and maximal [Ca^2+]^ but remained significantly faster than murine *k*tr values measured at similar levels of Ca^2+^. Once again, if we assume that Ca^2+^ binding occurs within randomly distributed RUs along the thin filament (Risi et al., 2021), then some regions of the thin filament will have a greater collection of activated RUs and other regions will have fewer numbers of activated RUs. Thus, the probability of activated RUs being in the near vicinity of other RUs in the closed or open state will be increased. As the [Ca^2+]^ rises to intermediate levels, the effective size of the functional group appears to increase as more activated RUs are incorporated. The central region of an activated functional group will have the greatest amounts of Ca^2+^ and crossbridges bound. Because of this, the rate of force redevelopment will be progressively slowed due to cooperative recruitment of crossbridges into the end regions of the functional group or adjacent functional groups.

As the [Ca^2+]^ continues to rise toward maximal levels, the number of activated RUs with Ca^2+^ bound increases in both murine and porcine myocardium. Thus, fewer inactivated RUs remain, and the concomitant impact of near-neighbor cooperative interactions will be reduced. As a result, the time course of force redevelopment in porcine and murine myocardium becomes progressively faster, and *k*tr converges to the respective values obtained during maximal activation.

### Evidence for the dynamic cooperative behavior of murine and porcine myocardium—the effect of the near-neighbor RU–RU cooperative interaction

**Figure 8.**
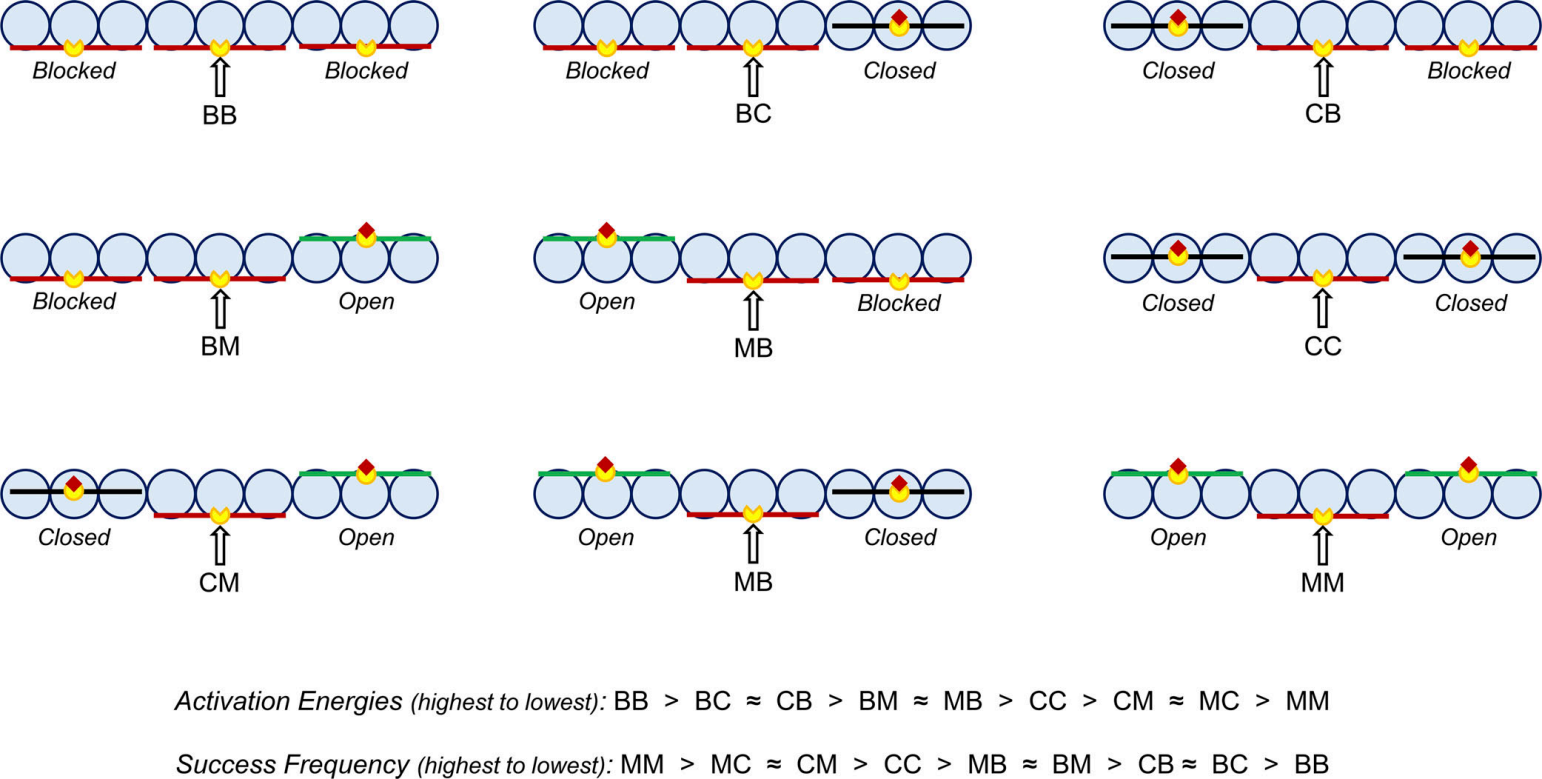
**Possible near neighbor effects on a thin filament regulatory unit.** The probability of a central RU transitioning from the blocked to closed to open state is influenced by the states of its neighboring RUs: both neighbors blocked (BB); one neighbor blocked, one neighbor closed (BC, CB); one neighbor blocked, one neighbor open (BM, MB); bothe neighbors closed (CC); one neighbor closed, one neighbor open (CM, MC); and both neighbors open (MM). The success frequency of a central RU transitioning away from the blocked state is highest when both neighbors are in the open state (i.e., activation energy is lowest) and lowest when both neighbors are in the blocked state (i.e., activation energy is the highest). Figure adapted from Razumova et al. (2000).

Here, we propose that the relative contribution of the ensemble near-neighbor RU–RU, XB–XB, and XB–RU cooperative interactions vary in a species-specific manner to modulate the activation dependence of the rate of force redevelopment. As a result of the model, two RU–RU cooperative coefficients (i.e., *u*_2_ and *z*_2_) were derived and shown to play a critical role in fitting the in vitro contractility data obtained in porcine and murine ventricular myocardium. Collectively, these cooperative coefficients demonstrate the dynamic near-neighbor cooperative behavior of mammalian myocardium. The cooperative coefficients *u*_2_ and *z*_2_ measure the strength of the near-neighbor RU–RU cooperative interaction, i.e., the degree of cooperative coupling between neighboring RUs. The cooperative coefficient *u*_2_ is closely related to the activation energy needed to be overcome for a central RU to make the transition from the blocked to the closed state. When *u*_2_ = 1, there is no cooperative RU–RU interaction. However, as the value of *u*_2_ increases (i.e., *u*_2_ > 1), a greater activation energy is encountered for the transition of a central RU from the blocked to the closed state (Kalda and Vendelin, 2020). In both the murine and porcine thin filament, a central RU located between two RUs in the blocked state (*BB*, Fig. 8) will have a lower success frequency of transitioning compared with a central RU located between two RUs in the closed state (*CC*, Fig. 8). But the greater value of *u*_2_ modeled for porcine myocardium compared with murine myocardium (e.g., *u*_2_ = 17.082 versus 2.265; Table 2) suggests that a higher activation energy exists between near-neighbor RUs, making it harder for these neighboring RUs in the porcine thin filament to successfully transition to the closed state. It has been proposed that the introduction of stronger cooperative coupling between near-neighboring RUs should effectively slow the time course for force redevelopment (Campbell, 1997; Razumova et al., 1999). In both the murine and porcine thin filament, the initial Ca^2+^ binding to TnC would activate a RU, causing it to transition from the blocked to the closed state. At very low Ca^2+^, this activated RU would be located among neighboring RUs positioned in the blocked state. The relative strength of near-neighbor RU–RU interactions will initially define the extent of cooperative spread along the thin filament due to Ca^2+^ and subsequent crossbridge binding within the activated RU (Moss et al., 2004, 2015). As such, stronger near-neighbor RU–RU interactions, as modeled for the porcine thin filament, will tend to hold adjacent RUs in the blocked state, whereas weaker RU–RU interactions, as in the murine thin filament, would permit adjacent RUs to transition to the closed state. Since the strength of the RU–RU cooperative interactions is related to activation energy (Razumova et al., 2000; Kalda and Vendelin, 2020), the higher the value of *u*_*2*_ the greater the activation energy barrier to cooperatively recruit neighboring RUs to the closed state. Thus, the neighboring RUs in the porcine thin filament will exhibit a lower success frequency of transitioning to the closed state. If the spread cooperative activation is limited to a shorter segment of the thin filament (e.g., within a single RU versus within two to three RUs) at very low [Ca^2+]^ (Gillis et al., 2007), then the effect of a strong cooperative coupling to slow the time course of force redevelopment will be minimized, resulting in rates of force redevelopment that approach values seen at maximal [Ca^2+]^ (Fig. 6 B and Fig. 7 B). In a similar manner to that proposed for *u*_2_, the cooperative parameter *z*_2_ is closely related to the activation energy needed to be overcome for a central RU to make the transition from the closed to the open state. In both the murine and porcine thin filament, a central RU located between two RUs in the closed state (*CC*, Fig. 8) will have a lower success frequency of transitioning to the open state compared with a central RU located between two RUs in the open state (*MM*, Fig. 8). As the value of *z*_2_ increases, the degree of cooperative coupling between neighboring RUs becomes greater. In our model, the greater value of *z*_2_ derived for murine myocardium compared with porcine myocardium (e.g., z_2_ = 1.0118 versus 1.000; Table 2) suggests that a slightly stronger cooperative interaction governs the transition of a central RU from the closed to the open state in the murine thin filament. This suggests that the murine myocardial thin filament requires a slightly greater number of strongly bound crossbridges for activation, making it the more cooperative of the two muscles in this regard. Further elucidation of the cooperative mechanisms underlying the activation dependence of *k*tr can be ascertained by examining the model-derived nearest neighbor interaction factors $\left(\alpha ,\bar{\alpha },\beta ,\text{and}\;\bar{\beta }\right)$. The extent to which the RU–RU and XB–RU interactions effect the *B*-to-*C* transition (i.e., *k*_*BC*_) can be described by *α* and 1 − *α*, while the *C*-to-*B* transition (i.e., *k*_*CB*_) can be described by $\bar{\alpha }$ and $1-\bar{\alpha }$. In a similar fashion, the effects of the RU–RU interaction and XB–XB interaction on the *C*-to-*M*_*1*_ transition (i.e., $k_{CM_{1}}$) can be described by *β* and 1 − *β*, while the *M*_*1*_-to-*C* transition (i.e., $k_{M_{1}C}$) can be described by $\bar{\beta }$ and $1-\bar{\beta }$. The nearest neighbor interaction factors for porcine myocardium $\left(\alpha ,\bar{\alpha },\beta ,\text{and}\;\bar{\beta }\right)=\left(1.0,0.8296,1.0,\text{and}\;1.0\right)$ indicate that the process of force redevelopment is predominantly modulated by RU–RU cooperative interactions with only a slight involvement of XB–RU interactions. However, the nearest neighbor interaction factors for murine myocardium $\left(\alpha ,\bar{\alpha },\beta ,\text{and}\;\bar{\beta }\right)=\left(0.9996,0.8897,0.9466,\text{and}\;0.1376\right)$ signify that while force redevelopment is largely modulated by the RU–RU interactions, there is also additional significant involvement of the near-neighbor XB–RU and XB–XB cooperative interactions.

### The effect of altering the RU–RU cooperative coefficients *u*_2_ and *z*_2_ on the activation dependence of the rate of force redevelopment

**Figure 9.**
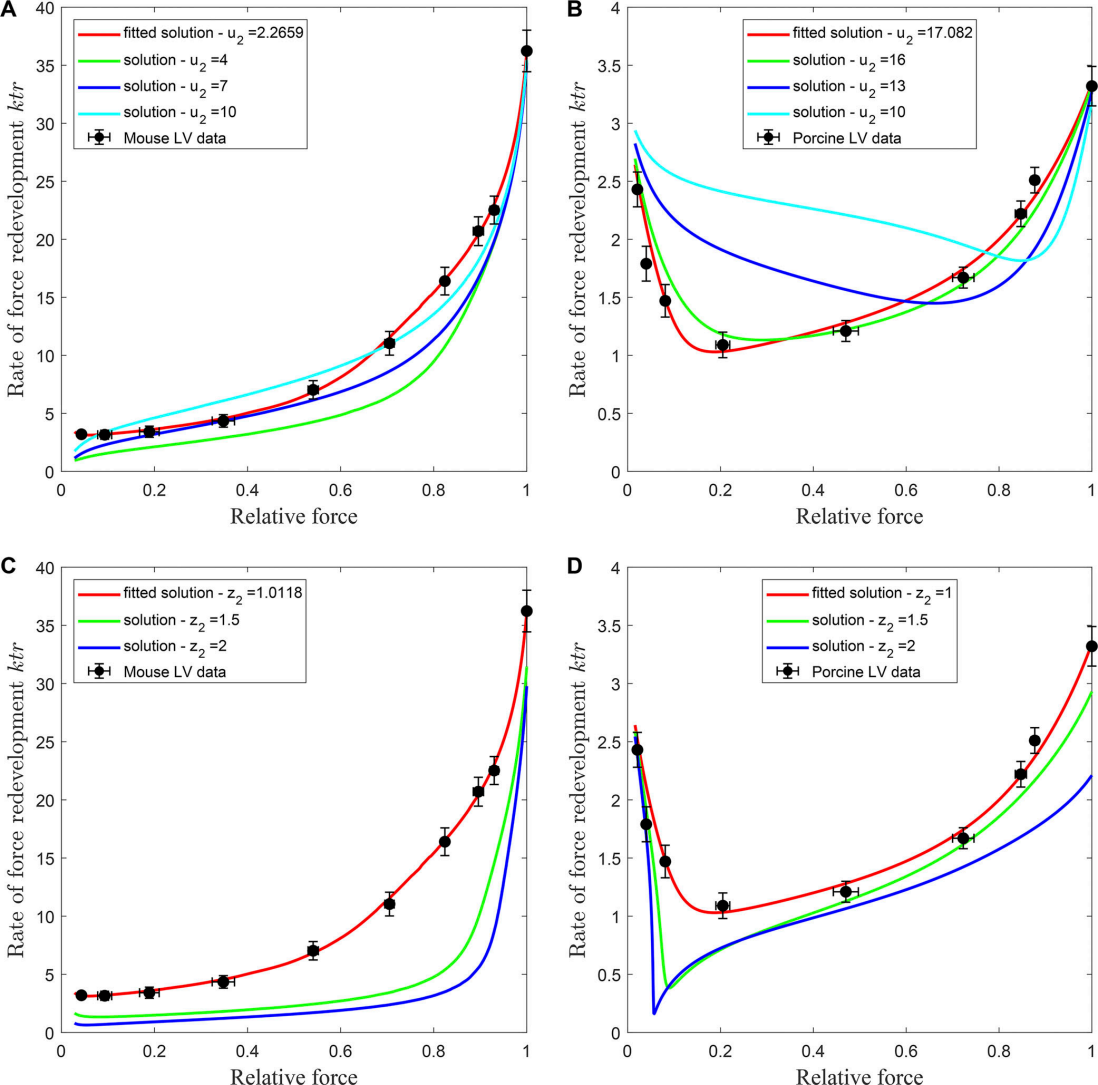
**The effects of altering the strength of RU-RU cooperative coefficients on the activation-dependencies of the rate of force development.** The rate constant of force redevelopment was measured in permeabilized myocardial preparations isolated from murine and porcine ventricular myocardium. The data points (filled circles) represent the means and the error bars are the SEM (from Table S1). The model, comprising Eqs. 2, 3, and 4, is fitted to the data of P/P_o_ versus pCa and the data of *ktr* versus pCa in murine and porcine myocardium using the parameters derived from the ensemble RU-RU, XB-XB, and XB-RU interactions (Parameter Set 8 - Table 4). **(A and B)** The effect of altering the RU-RU blocked-to-closed cooperative coefficient *u_2_* on the activation-dependence of *ktr* in murine (A) and porcine (B) myocardium. **(C and D)** The effect of altering the RU-RU closed-to-open cooperative coefficient *z_2_*on the activation-dependence of *ktr* in murine (C) and porcine (D) myocardium.

Our modeling data suggest that the responsiveness of the myocardial thin filament to the activating effects of Ca^2+^ and myosin crossbridges varies in a species-dependent manner. To further investigate the role of the RU–RU cooperative interactions on the contractile dynamics in murine and porcine myocardium, we modeled the effects of independently varying either *u*_*2*_ or *z*_*2*_ on the activation dependence of *k*tr (Fig. 9 and Table 4). By using Eqs. 15 and 16, we can define an “activation factor” as *K*, where *K* = *k*_BC_/*k*_CB_. Therefore, *K* becomes a function of the Ca^2+^ concentration, the RU–RU and XB–RU cooperative coefficients (*u*_*1*_, *u*_*2*_, and *w*), the RU–RU and XB–RU nearest neighbor interaction factors of the transitions between the *B* and *C* state (*α*, 1 − *α*, $\bar{\alpha }$, $1-\bar{\alpha }$), and the state variables *B*, *M*_*1*_, and *M*_*2*_, i.e., *K* = *K*(Ca^2+^, *u*_1_, *u*_2_, *w*, *α*, 1 − *α*, $\bar{\alpha }$, $1-\bar{\alpha }$, *B*, *M*_*1*_, and *M*_*2*_). Changes in *K* reflect the sensitivity of myocardial thin filament to the activating effects of Ca^2+^ binding when any of the cooperative coefficients (*u*_1_, *u*_2_, and *w*) are altered. By using Eqs. 17 and 18, we can define a “cross-bridge recruitment factor” as *N*, where *N* = *k*_CM1_/*k*_M1C_. Thus, *N* is a function of the RU–RU and XB–XB cooperative coefficients (*z*_*1*_, *z*_*2*_, and *v*), the RU–RU and XB–XB nearest neighbor interaction factors of the transitions between the *C* and *M*_*1*_ state (*β*, 1 − *β*, $\bar{\beta }$, $1-\bar{\beta }$), and the state variables *B*, *M*_*1*_, and *M*_*2*_, i.e., *N* = *N*(*z*_*1*_, *z*_*2*_, *v*, *β*, 1 − *β*, $\bar{\beta }$, $1-\bar{\beta }$, *B*, *M*_*1*_, and *M*_*2*_). Changes in *N* indicate the sensitivity of the myocardial thin filament to the activating effects of crossbridge binding when any of the cooperative coefficients (*z*_*1*_, *z*_*2*_, and *v*) are altered.

**Table 4.** Summary of effect of altering RU–RU cooperative coefficients *u*_2_ and *z*_2_ using ensemble parameter set 8

Murine LV: *α* = 0.9996, $\bar{\alpha }=0.8897$, *β* = 0.9466, and $\bar{\beta }=0.1376$																								
*u*_*1*_		1.0054				1.0054				1.0054				1.0054				1.0054				1.0054		
*u*_*2*_		**2.2659**				**4.0**				**7.0**				**10.0**				2.2659				2.2659		
*z*_*1*_		1.0047				1.0047				1.0047				1.0047				1.0047				1.0047		
*z*_*2*_		1.0118				1.0118				1.0118				1.0118				**1.50**				**2.00**		
*V*		1.0067				1.0067				1.0067				1.0067				1.0067				1.0067		
*W*		1.0463				1.0463				1.0463				1.0463				1.0463				1.0463		
pCa		6.1		4.5		6.1		4.5		6.1		4.5		6.1		4.5		6.1		4.5		6.1		4.5
K		4.13e-6		7.66e-6		1.48e-5		2.15e-5		5.42e-5		6.31e-5		1.15e-4		1.25e-4		5.57e-6		1.11e-5		9.85e-6		1.60e-5
N		0.8563		0.8654		0.8620		0.8659		0.8648		0.8661		0.8655		0.8661		1.1880		1.4687		2.1035		2.6516
B		0.4774		0.0367		0.2020		0.0131		0.0642		0.0046		0.0312		0.0023		0.3709		0.0196		0.1999		0.0093
C		0.2796		0.5129		0.4256		0.5253		0.4984		0.5298		0.5158		0.5310		0.2867		0.3972		0.2590		0.2376
M1		0.1665		0.3085		0.2551		0.3161		0.2996		0.3189		0.3103		0.3196		0.2346		0.3994		0.3707		0.4910
M2		0.0765		0.1419		0.1173		0.1455		0.1378		0.1467		0.1428		0.1471		0.1079		0.1838		0.1705		0.2260
*Ktr*		3.1937		36.1580		1.4919		35.2720		2.2195		35.1960		3.2629		35.4490		1.3378		31.4560		0.6910		29.7690
Rel *ktr*		0.0883		1.0		0.0423		1.0		0.0631		1.0		0.0920		1.0		0.0425		1.0		0.0232		1.0
Porcine LV: *α* = 1, $\bar{\alpha }=0.8296$, *β* = 1, and $\bar{\beta }=1$																								
*u*_1_	1.1055				1.1055				1.1055				1.1055				1.1055				1.1055			
*u*_2_	**17.082**				**16.0**				**13.0**				**10.0**				17.082				17.082			
*z*_1_	1.0001				1.0001				1.0001				1.0001				1.0001				1.0001			
*z*_2_	1.0				1.0				1.0				1.0				**1.50**				**2.00**			
*v*	1.0374				1.0374				1.0374				1.0374				1.0374				1.0374			
*w*	1.0				1.0				1.0				1.0				1.0				1.0			
pCa	6.1		4.5		6.1		4.5		6.1		4.5		6.1		4.5		6.1		4.5		6.1		4.5	
K	0.0203		0.3312		0.0190		0.2957		0.0064		0.0897		0.0058		0.0613		0.0211		0.6670		0.0220		2.5188	
N	0.1314		0.1315		0.1314		0.1315		0.1314		0.1315		0.1314		0.1315		0.1361		0.2033		0.1450		0.4776	
B	0.8998		0.0339		0.9055		0.0377		0.9179		0.0530		0.9268		0.0796		0.8954		0.0155		0.8910		0.0032	
C	0.0809		0.7794		0.0762		0.7763		0.0662		0.7640		0.0591		0.7425		0.0838		0.7184		0.0868		0.5332	
M_1_	0.0106		0.1024		0.0100		0.1020		0.0087		0.1000		0.0078		0.0976		0.0114		0.1459		0.0122		0.2543	
M_2_	0.0088		0.0843		0.0082		0.0840		0.0072		0.0827		0.0064		0.0803		0.0094		0.1202		0.0100		0.2094	
*k*tr	2.2918		3.3452		2.3927		3.3289		2.6257		3.2702		2.8114		3.1842		2.1604		2.9306		2.0321		2.2114	
Rel *k*tr	0.6851		1.0		0.7188		1.0		0.8029		1.0		0.8829		1.0		0.7372		1.0		0.9189		1.0	

Summary of computational results for the model fit of *k*tr-relative force (Table S1). The model is run with parameter set 8 with fitted rate coefficients taken from Table 4. Varying the strength of the RU-RU blocked-to-closed cooperative coefficient *u_2_* and the closed-to-open cooperative coefficient* z_2_* are highlighted in bold. The values of *B*, *C*, *M*_*1*_, and *M*_*2*_ are recorded when the solution of the ODE system Eqs. 2, 3, and 4 reach their equilibrium state. The activation factor (K = *k*_BC_/*k*_CB_) and crossbridge recruitment factor (N = *k*_CM1_/*k*_M1C_) are computed based on these values. Finally, *k*tr is computed according to the slack-restretch maneuver (Patel et al., 2023).

We modeled the effect of varying *u*_*2*_ on the blocked-to-closed RU transition using parameter set 8 with the cooperative coefficients *u*_*1*_, *z*_*1*_, *z*_*2*_, *v*, and *w* held constant. In murine myocardium, we examined the effects on the activation dependence of *k*tr by progressively strengthening the degree of cooperative coupling between near-neighbor RUs by increasing *u*_2_ (Fig. 9 A and Table 4). Increasing *u*_2_ from 2.2659 to 4.0 significantly steepened the activation dependence of *k*tr. The nearly 24-fold increase in *k*tr from low to maximal levels of Ca^2+^ activation was due to the positive cooperative feedback between *M*_*2*_ and the activation factor *K*. By Eqs. 15 and 16, since *α* = 0.9996 and $\bar{\alpha }=0.8897$, the relatively small increase in *u*_2_ increased the value of *K*, implying that the forward transition rate *k*_BC_ increases faster than the reverse transition rate *k*_CB_. This results in a larger number of crossbridges available for cooperative recruitment into cycling population, which increases *M*_*2*_ at all levels of Ca^2+^ activation. Because *z*_*1*_ and *z*_*2*_ (i.e., 1.0047 and 1.0118) are close to 1.0, the value of *N* remains constant as *u*_*2*_ is increased. In porcine myocardium, we modeled the effects of decreasing *u*_2_ from 17.082 to 10.0 (Fig. 9 B and Table 4), which results in a progressive weakening of the cooperative coupling between neighboring RUs. Because *z*_1_ = 1.0001 and *z*_2_ = 1.0, the value of crossbridge recruitment factor *N* remains constant (*N* = 0.1315) as *u*_*2*_ is decreased. Since *u*_1_ = 1.1055 (which is close to 1.0), the gradual decrease in *u*_2_ reduces the activation factor *K*, which decreases the value of *M*_2_, leading to a further decrease in *K*. This suggests there is negative feedback between the activation factor *K* and the force-generating crossbridge population *M*_*2*_, when *u*_2_ is reduced. As a result, the rate of force redevelopment at low and intermediate levels of Ca^2+^ activation increases, thereby reducing the activation dependence of *k*tr. The modeling result for porcine myocardium is intriguing when considered in the context of hypertrophic cardiomyopathy (HCM)-related mutations in Tm (Loong et al., 2012; Moore et al., 2016; Matyushenko et al., 2017, 2023; Tsaturyan et al., 2022; Berry et al., 2024) and troponin T (TnT) (Sommese et al., 2013; Wang et al., 2018; Landim-Vieira et al., 2023). In the human myocardial thin filament, structural changes induced by HCM-related mutations in either TnT or Tm could have a substantial impact on the responsiveness of the thin filament to the activating effects of Ca^2+^ and crossbridge binding. If the TnT or Tm HCM mutations reduce the cooperative coupling between neighboring RUs (i.e., decreasing the activation energy), the level of thin filament activation would be increased, resulting in a concomitant reduction in the activation dependence of *k*tr (Table 4).

To investigate the species specificity of the closed-to-open RU transition, we examined the effects of increasing *z*_2_ on the activation dependence of *k*tr. To do so, we used the ensemble parameter set 8 with all cooperative coefficients held constant and allowed *z*_*2*_ to increase. In both murine (Fig. 9 C) and porcine myocardium (Fig. 9 D), increasing *z*_*2*_ from ∼1.0 to 2.0 resulted in a significant decrease in *k*tr at low and intermediate levels of Ca^2+^ activation. This effect arises due to the positive feedback between *M*_*2*_ state and the crossbridge recruitment factor *N* as seen in Eqs. 17 and 18. Likewise, with the RU–RU and XB–RU cooperative coefficients (i.e., *u*_*1*_, *u*_*2*_, and *w*) held constant, Eqs. 15 and 16 imply that a slight increase in *M*_*2*_ will increase the activation factor *K*, resulting in a net movement of crossbridges from the non-cycling (*B*) to the cycling (*C*, *M*_*1*_, and *M*_*2*_) population. This will effectively reduce *k*tr as the [Ca^2+^] increases from threshold to maximal levels.

In summary, our mathematical model accurately fit the in vitro contractility data measured in murine- and porcine-permeabilized myocardium. Our model quantified the effects of near-neighbor interaction on the cooperative development of force, thereby allowing for elucidation of the contribution of the RU–RU, XB–XB, and XB–RU near-neighbor interactions in mammalian myocardium. Our modeling result demonstrated that in both murine and porcine thin filament, the RU–RU cooperative interaction was the predominant near-neighbor effect, consistent with that proposed previously (Campbell et al., 2010). From an energetic perspective, the near-neighbor interaction requiring the largest free energy change would be predicted to be the rate-limiting cooperative factor. Our results agree with the model of Kalda and Vendelin (2020), who proposed that the changes in free energy associated with the RU–RU interaction are approximately fivefold larger than that for the XB–XB interaction. Furthermore, our model revealed species-specific differences in the ensemble effects of the combined RU–RU, XB–XB, and XB–RU cooperative interactions. The species-specific differential aggregate contribution of near-neighbor cooperative mechanisms underlies the differences in myocardial contractile dynamics and confers a physiologic adaptation to synchronize ventricular contractility with intrinsic beat frequency and twitch kinetics.

### Model limitations

It is well established that cooperative mechanisms modulate both force development and force relaxation in mammalian cardiac muscle. As such, molecular cooperativity is not described by a single factor but rather is defined by a wide array of mechanisms operating from the molecular level to more globally across the sarcomere. Given the multivariant nature of cooperativity, we chose to simplify our modeling efforts to initially examine near-neighbor interactions (i.e., RU–RU, RU–XB, and XB–XB) to establish a baseline understanding of how these cooperative interactions modulate force development. Our sensitivity analysis, represented by resolution matrices, indicated that while our model is sensitive to both forward and reverse cooperative parameters, there is a slightly higher sensitivity to the forward parameters under our experimental conditions.

Furthermore, we readily acknowledge that additional forms of cooperativity most likely play significant roles in modulating cardiac contraction and relaxation. For example, our model did not examine cooperative mechanisms that exert sarcomere-wide effects (Dupuis et al., 2016; Mijailovich et al., 2021), including myofilament compliance and lattice geometry (Tanner et al., 2007, 2012), as well as communication between adjoining thin filaments (Risi et al., 2021). Furthermore, our model did not explicitly examine the contributions of the thin filament regulatory proteins Tm (Smith and Geeves, 2003; Campbell et al., 2010; Geeves et al., 2011; Moore et al., 2016; McConnell et al., 2017) and troponin (Farman et al., 2010; Kreutziger et al., 2011; Land and Niederer, 2015; Landim-Vieira et al., 2023) or the effects of phosphorylation of the myosin regulatory light chain (Toepfer et al., 2013; Karabina et al., 2015; Turner et al., 2023) and myosin-binding protein-C (Moss et al., 2015; Previs et al., 2016; Risi et al., 2022; Wong et al., 2024). The incorporation of these mechanisms, particularly the phosphorylation of cardiac-binding protein-C (cMyBP-C), is likely to significantly impact model outcomes and provide a more comprehensive understanding of the regulation of ventricular function. Cardiac-binding protein-C phosphorylation increases myocardial contractility (Stelzer et al., 2006b; Sadayappan et al., 2011; Gresham et al., 2017) at least in part, by promoting actin–MyBP-C interactions and augmenting the level of thin filament activation (Mun et al., 2014; Kampourakis et al., 2014; Risi et al., 2022; Wong et al., 2024), thereby potentially interacting with the near-neighbor cooperative interactions that we have modeled. While computational constraints necessitated our initial focus on near-neighbor cooperative interactions, we are actively working to refine our model. We recognize that the model-derived cooperative parameters provide a qualitative interpretation of the factors underlying myocardial contractile kinetics. Subsequent experimentation will be required to validate the quantitative contribution of these factors. To accomplish this, our immediate plans include incorporating relaxation transients into our experimental model, which will allow us to better explore mechanisms underlying cooperative inactivation of the thin filament. Subsequently, we aim to systematically integrate additional cooperative mechanisms, prioritizing those that our preliminary analyses suggest may have the most significant impact on force development and relaxation kinetics. Working with the framework of near-neighbor cooperative interactions, we will continually refine our model to incorporate additional cooperative mechanisms, particularly given how these mechanisms may be altered in disease states, such as cardiomyopathy (Sewanan et al., 2016; Farman et al., 2018; Mertens et al., 2024). We aim to enhance its predictive power and provide more comprehensive insights into cardiac function in health and disease.

## Supplementary Material

Table S1 shows the summary of steady-state mechanical data in murine- and porcine-permeabilized ventricular myocardium.

Table S2 shows the summary of state variables and model equations.

Table S3 shows the summary of rate coefficients, cooperative coefficients, and nearest neighbor interaction factors.

Table S4 shows the fitted parameters for modeling force–pCa relationships in murine- and porcine-permeabilized ventricular myocardium.

Table S5 shows the parameter identification for model resolution matrices.

Data S1 shows the steady-state contractile measurements.

## Data Availability

All in vitro contractility data underlying this study are available in the published article and its online supplemental material. The MATLAB code for our model has been deposited in Github: (https://github.com/tphan86/TF_model).
