# Supplementary material for: Modeling the effects of thin filament near-neighbor cooperative interactions in mammalian myocardium
Source: J Gen Physiol. 2025 Jan 27;157(2):e202413582. doi: 10.1085/jgp.202413582 (PMC11771317; doi:10.1085/jgp.202413582)
Supplement: Table S1 — shows the summary of steady-state mechanical data in murine- and porcine-permeabilized ventricular myocardium. [file jgp_202413582_tables1.docx]

**Table S1: Summary of Steady-State Mechanical Data in Murine and Porcine Permeabilized Ventricular Myocardium**

|  | ***Murine Myocardium*** | | |
| --- | --- | --- | --- |
| **pCa** | **P/P_o_** | ***k*tr** | **Relative *k*tr** |
| 6.2 | 0.043 ± 0.006 | 3.20 ± 0.28 | 0.093 ± 0.009 |
| 6.1 | 0.093 ± 0.015 | 3.16 ± 0.39 | 0.096 ± 0.013 |
| 6.0 | 0.189 ± 0.021 | 3.42 ± 0.48 | 0.103 ± 0.013 |
| 5.9 | 0.348 ± 0.024 | 4.35 ± 0.54 | 0.135 ± 0.015 |
| 5.8 | 0.541 ± 0.011 | 7.03 ± 0.79 | 0.225 ± 0.022 |
| 5.7 | 0.705 ± 0.010 | 11.04 ± 1.02 | 0.367 ± 0.026 |
| 5.6 | 0.824 ± 0.007 | 16.40 ± 1.19 | 0.569 ± 0.029 |
| 5.5 | 0.896 ± 0.011 | 20.70 ± 1.24 | 0.750 ± 0.029 |
| 5.4 | 0.930 ± 0.009 | 22.52 ± 1.20 | 0.851 ± 0.020 |
| 4.5 | 1.0 ± 0.0 | 36.23 ± 1.79 | 1.0 ± 0.0 |
|  |  |  |  |
|  | ***Porcine Myocardium*** | | |
| **pCa** | **P/P_o_** | ***k*tr** | **Relative *k*tr** |
| 6.2 |  |  |  |
| 6.1 | 0.021 ± 0.003 | 2.43 ± 0.15 | 0.742 ± 0.034 |
| 6.0 | 0.040 ± 0.005 | 1.79 ± 0.15 | 0.548 ± 0.035 |
| 5.9 | 0.081 ± 0.008 | 1.47 ± 0.14 | 0.443 ± 0.023 |
| 5.8 | 0.205 ± 0.015 | 1.09 ± 0.11 | 0.333 ± 0.019 |
| 5.7 | 0.470 ± 0.027 | 1.21 ± 0.09 | 0.377 ± 0.013 |
| 5.6 | 0.723 ± 0.023 | 1.67 ± 0.09 | 0.535 ± 0.015 |
| 5.5 | 0.847 ± 0.012 | 2.22 ± 0.11 | 0.715 ± 0.012 |
| 5.4 | 0.877 ± 0.008 | 2.51 ± 0.11 | 0.817 ± 0.012 |
| 4.5 | 1.0 ± 0.0 | 3.32 ± 0.17 | 1.0 ± 0.0 |

All values are expressed as means ± S.E.M. P/P_o_, relative Ca^2+^-activated force determined by expressing submaximal force (P) at each pCa as a fraction of maximal force (P_o_) measured at pCa 4.5; *k*tr, rate constant of force redevelopment in seconds^-1^; relative *k*tr, the submaximal rate of force redevelopment normalized to the maximal rate of force redevelopment measured at pCa 4.5. *In vitro* mechanical data for murine myocardium was collected *de novo*, while that for porcine ventricular myocardium was collected previously (Patel et al., 2023).

**Refences**

Patel, J.R., K.J.V. Park, A.S. Bradshaw, T. Phan, and D.P. Fitzsimons. 2023. Cooperative mechanisms underlie differences in myocardial contractile dynamics between large and small mammals. *J. Gen. Physiol.* 155:e2022133315.
