# Supplementary material for: Modeling the effects of thin filament near-neighbor cooperative interactions in mammalian myocardium
Source: J Gen Physiol. 2025 Jan 27;157(2):e202413582. doi: 10.1085/jgp.202413582 (PMC11771317; doi:10.1085/jgp.202413582)
Supplement: Table S2 — shows the summary of state variables and model equations. [file jgp_202413582_tables2.docx]

**Table S2. Summary of State Variables and Model Equations**

| *State Variables* | |
| --- | --- |
| *B* | The fraction of RUs in the blocked state; or  The fraction of XBs that are in the unbound state |
| *C* | The fraction of RUs in the closed state; or  The fraction of XBs in the weakly bound state |
| *M_1_* | The fraction of RUs that are in the open *M_1_* state; or  The fraction of XBs that are in the strongly bound non-force generating state |
| *M_2_* | The fraction of RUs that are in the open *M_2_* state; or  The fraction of XBs that are in the strongly bound force-generating state |
| *Model Equations* | |
| *Eq. 1* | $\dot{B}=k_{CB}C-k_{BC}B$ |
| *Eq. 2* | $\dot{C}=k_{BC}B+k_{M_{2}C}M_{2}+k_{M_{1}C}M_{1}-(k_{CB}+k_{CM_{1}})C$ |
| *Eq. 3* | $\dot{M}_{1}=k_{CM_{1}}C+k_{M_{2}M_{1}}M_{2}-(k_{M_{1}C}+k_{M_{1}M_{2}})M_{1}$ |
| *Eq. 4* | $\dot{M}_{2}=k_{M_{1}M_{2}}M_{1}-(k_{M_{2}C}+k_{M_{2}M_{1}})M_{2}$ |
| *Eq. 5* | $k_{BC}=k_{BC}^{0}+[k_{BC}^{\text{C}\text{a}^{2+}}-k_{BC}^{0}]\frac{\text{C}\text{a}^{2+}}{\text{C}\text{a}_{50}^{2+}+\text{C}\text{a}^{2+}}$ |
| *Eq. 6* | $k_{CB}=k_{CB}^{0}+[k_{CB}^{\text{C}\text{a}^{2+}}-k_{CB}^{0}]\frac{\text{C}\text{a}^{2+}}{\text{C}\text{a}_{50}^{2+}+\text{C}\text{a}^{2+}}$ |
| *Eq. 7* | $k_{BC}:=k_{BC}^{(u_{1},u_{2})}=k_{BC}^{(1,1)}\left[ 1-\lambda^{B}(1-u_{1}^{-1})+\lambda^{M}(u_{2}-1) \right]^{2}$ |
| *Eq. 8* | $k_{CB}:=k_{CB}^{(u_{1},u_{2})}=k_{CB}^{(1,1)}\left[ 1+\lambda^{B}(u_{1}-1)-\lambda^{M}(1-u_{2}^{-1}) \right]^{2}$ |
| *Eq. 9* | $k_{CM_{1}}:=k_{CM_{1}}^{(z_{1},z_{2})}=k_{CM_{1}}^{(1,1)}\left[ 1-\lambda^{B}(1-z_{1}^{-1})+\lambda^{M}(z_{2}-1) \right]^{2}$ |
| *Eq. 10* | $k_{M_{1}C}:=k_{M_{1}C}^{(z_{1},z_{2})}=k_{M_{1}C}^{(1,1)}\left[ 1+\lambda^{B}(z_{1}-1)-\lambda^{M}(1-z_{2}^{-1}) \right]^{2}$ |
| *Eq. 11* | $k_{CM_{1}}:=k_{CM_{1}}^{v}=f_{CM_{1}}^{0}\left[ 1+\lambda^{M_{2}}(e^{v-1}-1) \right]^{2}$ |
| *Eq. 12* | $k_{M_{1}C}:=k_{M_{1}C}^{v}=f_{M_{1}C}^{0}\left[ 1+\lambda^{M_{2}}(e^{-v+1}-1) \right]^{2}$ |
| *Eq. 13* | $k_{BC}:=k_{BC}^{w}=f_{BC}^{0}\left[ 1+\lambda^{M_{2}}(e^{w-1}-1) \right]^{2}$ |
| *Eq. 14* | $k_{CB}:=k_{CB}^{w}=f_{CB}^{0}\left[ 1+\lambda^{M_{2}}(e^{-w+1}-1) \right]^{2}$ |
| *Eq. 15* | $k_{BC}=f_{BC}^{0}\left\{ \alpha\left[ 1-\lambda^{B}(1-u_{1}^{-1})+\lambda^{M}(u_{2}-1) \right]^{2}+(1-\alpha)\left[ 1+\lambda^{M_{2}}(e^{w-1}-1) \right]^{2} \right\}$ |
| *Eq. 16* | $k_{CB}=f_{CB}^{0}\left\{ \bar{\alpha}\left[ 1+\lambda^{B}(u_{1}-1)-\lambda^{M}(1-u_{2}^{-1}) \right]^{2}+(1-\bar{\alpha})\left[ 1+\lambda^{M_{2}}(e^{-w+1}-1) \right]^{2} \right\}$ |
| *Eq. 17* | $k_{CM_{1}}=f_{CM_{1}}^{0}\left\{ \beta\left[ 1-\lambda^{B}(1-z_{1}^{-1})+\lambda^{M}(z_{2}-1) \right]^{2}+(1-\beta)\left[ 1+\lambda^{M_{2}}(e^{v-1}-1) \right]^{2} \right\}$ |
| *Eq. 18* | $k_{M_{1}C}=f_{M_{1}C}^{0}\left\{ \bar{\beta}\left[ 1+\lambda^{B}(z_{1}-1)-\lambda^{M}(1-z_{2}^{-1}) \right]^{2}+(1-\bar{\beta})\left[ 1+\lambda^{M_{2}}(e^{-v+1}-1) \right]^{2} \right\}$ |
