# Supplementary material for: Modeling the effects of thin filament near-neighbor cooperative interactions in mammalian myocardium
Source: J Gen Physiol. 2025 Jan 27;157(2):e202413582. doi: 10.1085/jgp.202413582 (PMC11771317; doi:10.1085/jgp.202413582)
Supplement: Table S3 — shows the summary of rate coefficients, cooperative coefficients, and nearest neighbor interaction factors. [file jgp_202413582_tables3.docx]

**Table S3. Summary of Rate Coefficients, Cooperative Coefficients, and Nearest Neighbor Interaction Factors**

| *Rate Coefficients* | |
| --- | --- |
| pCa_50_ | [Ca^2+^] required for half maximal activation |
| *k*^0^_BC_ | The *B*-to-*C* transition rate of an RU in the absence of Ca^2+^ |
| *k*^Ca2+^_BC_ | The *B*-to-*C* transition rate of an RU transitions in the presence of Ca^2+^ |
| *k*^0^_CB_ | The *C*-to-*B* transition of an RU transitions in the absence of Ca^2+^ |
| *k*^Ca2+^_CB_ | The *C*-to-*B* transition rate of an RU transitions in the presence of Ca^2+^ |
| *k^(1,1)^_BC_ = f*^0^_BC_ | The *B*-to-*C* transition rate of an RU with no near-neighbor interactions;  The XB transition rate from unbound to weakly bound with no near neighbor interactions |
| *k^(1,1)^_CB_ = f*^0^_CB_ | The *C*-to-*B* transition rate of an RU with no near-neighbor interactions;  The XB transition rate from weakly bound to unbound with no near neighbor interactions |
| *f*^0^_CM1_ | The *C*-to-*M_1_* transition rate of an RU with no near-neighbor interactions;  The XB transition rate from weakly to strongly bound non-force generating state with no near neighbor interactions |
| *f*^0^_M1C_ | The *M_1_*-to-*C* transition rate of an RU with no near-neighbor interactions;  The XB transition rate from strongly bound non-force generating state to weakly bound with no near neighbor interactions |
| *k*_M1M2_ | The *M_1_*-to-*M_2_* transition rate of an RU;  The XB transition rate from non-force generating to force-generating strongly bound state |
| *k*_M2M1_ | The *M_2_*-to-*M_1_* transition rate of an RU;  The XB transition rate from force generating to non-force generating strongly bound state |
| *k*_M2C_ | The *M_2_*-to-*C* transition rate of an RU |
| *Cooperative Coefficients* | |
| *u_1_* | Strength of RU-RU interactions on the transition rate $k_{CB}$ |
| *u_2_* | Strength of RU-RU interactions on the transition rate $k_{BC}$ |
| *z_1_* | Strength of RU-RU interactions on the transition rate $k_{M_{1}C}$ |
| *z_2_* | Strength of RU-RU interactions on the transition rate $k_{CM_{1}}$ |
| *v* | Strength of XB-XB interactions on the transition rates $k_{CM_{1}}$ and $k_{M_{1}C}$ |
| *w* | Strength of XB-RU interactions on the transition rates $k_{BC}$ and $k_{CB}$ |
| *Nearest Neighbor Interaction Factors* | |
| $\alpha$ | Extent to which RU-RU interactions effect the transition rate $k_{BC}$ |
| $1-\alpha$ | Extent to which XB-RU interactions effect the transition rate $k_{BC}$ |
|  |  |
| $\bar{\alpha}$ | Extent to which RU-RU interactions effect the transition rate $k_{CB}$ |
| $1-\bar{\alpha}$ | Extent to which XB-RU interactions effect the transition rate $k_{CB}$ |
|  |  |
| $\beta$ | Extent to which RU-RU interactions effect the transition rate $k_{CM_{1}}$ |
| $1-\beta$ | Extent to which XB-XB interactions effect the transition rate $k_{CM_{1}}$ |
|  |  |
| $\bar{\beta}$ | Extent to which RU-RU interactions effect the transition rate $k_{M_{1}C}$ |
| $1-\bar{\beta}$ | Extent to which XB-XB interactions effect the transition rate $k_{M_{1}C}$ |
