# Supplementary material for: Modeling the effects of thin filament near-neighbor cooperative interactions in mammalian myocardium
Source: J Gen Physiol. 2025 Jan 27;157(2):e202413582. doi: 10.1085/jgp.202413582 (PMC11771317; doi:10.1085/jgp.202413582)
Supplement: Table S4 — shows the fitted parameters for modeling force–pCa relationships in murine- and porcine-permeabilized ventricular myocardium. [file jgp_202413582_tables4.docx]

**Table S4: Fitted Parameters for Modeling Force-pCa Relationships in Murine and Porcine Permeabilized Ventricular Myocardium**

|  | Murine Parameter Sets | | | Porcine Parameter Sets | | |
| --- | --- | --- | --- | --- | --- | --- |
| Parameter | Set 1 | Set 2 | Set 8 | Set 1 | Set 2 | Set 8 |
| pCa_50_ | 5.79 | 5.79 | 5.79 | 5.71 | 5.65 | 5.68 |
| *k*^0^_BC_ | 0.0 | 0.0 | 0.0 | 1.45E-08 | 0.0 | 0.0 |
| *k*^Ca2+^_BC_ | 16.2353 | 7.8858 | 7.9103 | 7.2767 | 1.3430 | 1.2527 |
| *k*^0^_CB_ | 19.4695 | 16.5774 | 16.5296 | 19.5205 | 9.4252 | 9.4176 |
| *k*^Ca2+^_CB_ | 0.6911 | 7.0281 | 7.0321 | 5.5975 | 0.0 | 0.0 |
| *f*^0^_CM1_ | 0.7777 | 0.1832 | 0.1950 | 0.6616 | 0.4765 | 0.5069 |
| *f*^0^_M1C_ | 6.2043 | 2.5028 | 2.5399 | 16.7590 | 2.9887 | 2.9407 |
| *k*_M1M2_ | 20.6862 | 5.2933 | 5.2742 | 11.5192 | 3.7358 | 3.7426 |
| *k*_M2M1_ | 18.3586 | 11.3259 | 11.3268 | 3.7274 | 5.6719 | 5.6627 |
| *k*_M2C_ | 14.3086 | 0.2915 | 0.2935 | 13.8142 | 0.1828 | 0.1226 |
| *u*_1_ | 1.0 | 1.0 | 1.0097 | 1.0 | 1.0 | 1.0472 |
| *u*_2_ | 1.0 | 17.5408 | 17.5650 | 1.0 | 14.1755 | 14.4390 |
| *z*_1_ | 1.0 | 1.0 | 1.0005 | 1.0 | 1.0 | 1.0 |
| *z*_2_ | 1.0 | 4.4995 | 4.5102 | 1.0 | 2.2877 | 2.2258 |
| *v* | 1.0 | 1.0 | 1.0005 | 1.0 | 1.0 | 1.0473 |
| *w* | 1.0 | 1.0 | 1.0024 | 1.0 | 1.0 | 1.0 |
| α | 1.0 | 1.0 | 0.9433 | 1.0 | 1.0 | 1.0 |
| α | 1.0 | 1.0 | 0.8914 | 1.0 | 1.0 | 0.7403 |
| β | 1.0 | 1.0 | 0.9246 | 1.0 | 1.0 | 0.9165 |
| β | 1.0 | 1.0 | 0.9974 | 1.0 | 1.0 | 0.9410 |
| RMSE | 0.5534 | 0.0076 | 0.0076 | 0.6184 | 0.0309 | 0.0309 |

Fitted parameters for force-pCa relationships in murine and porcine permeabilized myocardium using Parameter Set 1 (no near neighbor cooperative interaction), Parameter Set 2 (describing the unitary effect of the RU-RU cooperative interaction), and Parameter Set 8 (describing the ensemble effects of RU-RU, XB-XB, and XB-RU cooperative interactions). The cooperative parameters *u*_2_ and *w* measure the strength of the effects of RU-RU and XB-RU interactions on the activation factor K = *k*_BC_/*k*_CB_, respectively. The cooperative parameters *z_2_* and *v* measure the strength of the effects of RU-RU and XB-XB interactions on the cross-bridge recruitment factor *N* = *k*_CM1_/*k*_M1C_, respectively. Note that parameters α, α, β, and β measure the extent of the effects of RU-RU interaction on the system α = RU-RU_BC_; 1-α = XB-RU_BC_; α = RU-RU_CB_; 1-α = XB-RU_CB_; β = RU-RU_CM1_; 1-β = XB-XB_CM1_; β = RU-RU_M1C_; 1-β = XB-XB_M1C_.
