# Supplementary material for: Modeling the effects of thin filament near-neighbor cooperative interactions in mammalian myocardium
Source: J Gen Physiol. 2025 Jan 27;157(2):e202413582. doi: 10.1085/jgp.202413582 (PMC11771317; doi:10.1085/jgp.202413582)
Supplement: Table S5 — shows the parameter identification for model resolution matrices. [file jgp_202413582_tables5.docx]

**Table S5: Parameter Identification for Model Resolution Matrices**

|  | Parameter Sets | | |
| --- | --- | --- | --- |
| Parameter # | Set 1 | Set 2 | Set 8 |
| 1 | pCa_50_ | pCa_50_ | pCa_50_ |
| 2 | *k*^0^_BC_ | *k*^0^_BC_ | *k*^0^_BC_ |
| 3 | *k*^Ca2+^_BC_ | *k*^Ca2+^_BC_ | *k*^Ca2+^_BC_ |
| 4 | *k*^0^_CB_ | *k*^0^_CB_ | *k*^0^_CB_ |
| 5 | *k*^Ca2+^_CB_ | *k*^Ca2+^_CB_ | *k*^Ca2+^_CB_ |
| 6 | *f*^0^_CM1_ | *f*^0^_CM1_ | *f*^0^_CM1_ |
| 7 | *f*^0^_M1C_ | *f*^0^_M1C_ | *f*^0^_M1C_ |
| 8 | *k*_M1M2_ | *k*_M1M2_ | *k*_M1M2_ |
| 9 | *k*_M2M1_ | *k*_M2M1_ | *k*_M2M1_ |
| 10 | *k*_M2C_ | *k*_M2C_ | *k*_M2C_ |
| 11 |  | *u*_1_ | *u*_1_ |
| 12 |  | *u*_2_ | *u*_2_ |
| 13 |  | *z*_1_ | *z*_1_ |
| 14 |  | *z*_2_ | *z*_2_ |
| 15 |  |  | *v* |
| 16 |  |  | *w* |
| 17 |  |  | α |
| 18 |  |  | α |
| 19 |  |  | β |
| 20 |  |  | β |

Numerical identification of rate coefficients, cooperative coefficients (*u_1_*, *u_2_*, *z_1_*, *z_2_*, *v*, and *w*), and nearest neighbor interaction factors (α, α, β, and β) to evaluate the reliability of the model using model resolution matrices (Fig. S3).
