# Supplementary material for: Modeling the effects of thin filament near-neighbor cooperative interactions in mammalian myocardium
Source: J Gen Physiol. 2025 Jan 27;157(2):e202413582. doi: 10.1085/jgp.202413582 (PMC11771317; doi:10.1085/jgp.202413582)
Supplement: Data S1 — shows the steady-state contractile measurements. [file jgp_202413582_datas1.pdf]

SUPPLEMENT

I. Steady-State Contractile Measurements

Preparation of Murine Ventricular Tissue – Ten 129S1/Svlmj mice of either sex (3-6 mo old; The Jackson Laboratory) were injected with 5,000 U heparin / kg body weight and euthanized with isoflurane. Each heart was rapidly excised and placed in Ca^{2+}-free Ringer's solution (in mM: 118 NaCl, 25 HEPES, 11 glucose, 4.8 KCl, 1.2 Na_2HPO_4, and 1.2 $MgSO_4$, pH 7.4 and 22°C). The ventricles were separated at the septum and subsequently snap frozen in liquid N_2 and stored at -80°C until used. All procedures for murine care, handling and use were reviewed and approved by the University of Idaho Animal Care and Use Committee. On the day of use, frozen ventricular tissue were thawed in ice-cold relaxing solution and subsequently homogenized for 1-2 seconds using a Polytron homogenizer. The homogenates were centrifuged at 120 *x g* for 2 minutes. The pelleted multicellular preparations were permeabilized following resuspension in ice-cold relaxing solution containing 1% Triton-X100 (and 250 μg/ml saponin for murine samples) for 30 minutes. The preparations were washed 2X in fresh, ice-cold relaxing solution and stored on ice prior to mechanical measurements.

Solutions – Solution compositions were calculated using the computer program of Fabiato (1988) and stability constants (Godt and Lindley, 1982) corrected to pH 7.0, 22°C and 180 mM ionic strength using potassium propionate. The composition of solutions (in mM) are as follows; (1) relaxing solution: 100 KCl, 20 imidazole, 4 MgATP, 2 ethylene glycol-bis(2-aminoethyl ether)-*N,N,N',N'*-tetraacetic acid (EGTA), and 1 free Mg^{2+}; (2) pre-activating solution: 100 N,N-bis(2-hydroxyethyl)-2-aminoethanesulfonic acid (BES), 15 creatine phosphate (CP), 5 dithiothreitol (DTT), 4 MgATP, 1 free Mg^{2+}, and 0.07 EGTA; and (3) Ca^{2+}-activating solutions: 100 BES, 15 CP, 7 EGTA, 5 DTT, 4 MgATP, 1 free Mg^{2+}, with $[Ca^{2+}]_{free}$

ranging from 1 nM (i.e., pCa 9.0) to 32 μM (i.e., pCa 4.5). A range of submaximal pCa solutions containing differing $[Ca^{2+}]_{free}$ were prepared by mixing appropriate volumes of pCa 9.0 and pCa 4.5 solutions.

Steady-state mechanical measurements – The rate constant of force redevelopment (*k*tr) in skinned murine myocardium was assessed, as previously described (Giles, et. al., 2019). Each permeabilized preparation was transferred from pCa 9.0 solution to pre-activating solution and then into activating solutions of varying pCa (i.e., pCa 6.8 – 4.5) and allowed to develop steady-state isometric force. The preparation was rapidly (< 2 msec) slackened by 20% of its original length, followed by a brief period of unloaded shortening, after which the preparation was restretched rapidly (< 2 msec) to its initial length. A *k*tr-pCa relationship was obtained by first maximally activating a skinned myocardial preparation in a solution of pCa 4.5 and then in a series of submaximally activating solutions between pCa 6.8 – 5.4. To account for any run-down in maximal *k*tr, the myocardial preparation was activated in a solution of pCa 4.5 at the end of each experiment. The apparent rate constants of force redevelopment were estimated by linear transformation of the half-time of force redevelopment, i.e., $k\text{tr} = 0.693 / t_{1/2}$ (Giles et. al., 2019). During the measurements of the rate of force redevelopment, each myocardial preparation was bathed in solutions of varying pCa and allowed to develop steady-state isometric force. Total force at a specific pCa was calculated as the difference between steady-state force and the force baseline after the 20% slack step. Ca^{2+}-activated force at a given pCa was calculated as the difference between total force and Ca^{2+}-independent force measured in a solution of pCa 9.0 (Fig S1). All experiments were performed at 22°C and at an initial sarcomere length of ~ 2.20 μm in pCa 9.0 solution. **Absolute specific force (mN mm^{-2}) was calculated by assuming the permeabilized**

ventricular myocardial preparations were cylindrical, and by equating width obtained from video images of the mounted preparations to diameter (Chen et. al., 2010; Patel et. al., 2017**).** Changes in force and length-controller position were sampled (16-bit resolution, DAP5216a; Microstar Laboratories) at 2.0 kHz using SLControl software. All data were saved to computer files for data analysis. ***In vitro* contractility measurements of specific tension, relative force (*P/P*o), *k*tr and relative *k*tr (i.e., *k*tr$_{submax}$/*k*tr$_{max}$) values collected from murine and porcine permeabilized left ventricular myocardium are presented in Figures S1 and S2.** Table S1 **summarizes the values of relative Ca^{2+}-activated force (P/Po), the Ca^{2+}-dependencies of the rate of force redevelopment (*k*tr) and relative *k*tr.**

II. Detailed Presentation of the Mathematical Model

Our model contains a series of four differential equations:

$$\dot{B} = k_{CB}C - k_{BC}B, \tag{Eq. 1}$$

$$\dot{C} = k_{BC}B + k_{M_2C}M_2 + k_{M_1C}M_1 - (k_{CB} + k_{CM_1})C, \tag{Eq. 2}$$

$$\dot{M}_1 = k_{CM_1}C + k_{M_2M_1}M_2 - (k_{M_1C} + k_{M_1M_2})M_1, \tag{Eq. 3}$$

$$\dot{M}_2 = k_{M_1M_2}M_1 - (k_{M_2C} + k_{M_2M_1})M_2. \tag{Eq. 4}$$

From assumption 10, in which the total number of actin-myosin binding sites is fixed (R_T), $B + C + M_1 + M_2 = R_T$. Here, we denote *B* as the number of RUs that are in the blocked state, *C* as the number of RUs that are in the closed state along the thin filament. M_1 and M_2 are referred to as the number of RUs that are in the M_1 and M_2 state, respectively. A system of three differential equations (***Eqs.* 2, 3** and **4**) is sufficient to describe the rate of change of states in our 4-state model. The rate constants k_{BC} and k_{CB} describing the transitions between state *B* and state *C* depend on calcium concentration as in the following,

$$k_{BC} = k_{BC}^{0} + [k_{BC}^{\mathrm{Ca}^{2+}} - k_{BC}^{0}]\frac{\mathrm{Ca}^{2+}}{\mathrm{Ca}_{50}^{2+} + \mathrm{Ca}^{2+}}, \quad \textbf{\textit{(Eq. 5)}}$$

$$k_{CB} = k_{CB}^{0} + [k_{CB}^{\mathrm{Ca}^{2+}} - k_{CB}^{0}]\frac{\mathrm{Ca}^{2+}}{\mathrm{Ca}_{50}^{2+} + \mathrm{Ca}^{2+}}, \quad \textbf{\textit{(Eq. 6)}}$$

in which Ca_{50}^{2+} is the calcium concentration of thin filament binding sites at which the ratio in two above formulas equals 0.5. We only consider conditions of constant Ca^{2+} activation. By assumption 5, we also regard *B* as the number of XBs that are in the unbounded state, *C* as the number of XBs that are in the weakly bound state. M_1 and M_2 are also considered as the number of XBs that are in the strongly bound non-force generating state and in the strongly bound force generating state, respectively. Notice that, in some subsections below, we sometimes utilize *M* to indicate that an RU is in open state or a XB is in either M_1 or M_2 state.

The total cross-bridge population is divided into two subpopulations of non-cycling (State *B*) and cycling cross-bridges (i.e., States *C*, M_1, and M_2). State M_2 is the unique state that can generate force during isometric conditions and hence, isometric muscle force is proportional to the number of cycling cross-bridges in the M_2 state.

Each state and the combinations of states may be expressed as a fraction of R_T:

$\lambda^{B} = \frac{B}{R_T}$ - fraction of RUs that are blocked or fraction of XBs that are in the unbounded state.

$\lambda^{C} = \frac{C}{R_T}$ - fraction of RUs that are closed or fraction of XBs that are in the weakly bound state.

$\lambda^{M} = \frac{M_1 + M_2}{R_T}$ - fraction of RUs that are open or fraction of XBs that are either in M_1 or M_2 state.

$\lambda^{M_1} = \frac{M_1}{R_T}$ - fraction of RUs that are in M_1 state or fraction of XBs that are in the strongly bound non-force generating state.

$\lambda^{M_2} = \frac{M_2}{R_T}$ - fraction of RUs that are in M_2 state or fraction of XBs that are in the strongly bound force generating state.

Due to assumption 9, the above fractions represent the probability that a given RU or XB is in any one state. For simplicity, from now on we assume that R_T = 1. Hence

$\lambda^B = B$, $\lambda^C = C$, $\lambda^{M_1} = M_1$ and $\lambda^{M_2} = M_2$.

Below, we delineate in details how nearest neighbor RU-RU, XB-XB, and XB-RU interactions affects the transition rates k_{BC}, k_{CB}, k_{CM_1} and k_{M_1C} and the subsequent combination of all three nearest neighbor interactions into our thin filament model. A summary of state variables and model equations are presented in Table S2, while transitions rate coefficients, near neighbor cooperative coefficients, and nearest neighbor interaction factors are summarized in Table S3.

A. RU-RU interactions

We label the blocked state as either *B* or 0, the closed state as either *C* or 1, and the open state as either *M* or 2. The open state *M* is partitioned into strongly bound M_1 and M_2 states. Regulatory units (RUs), spanning 7 actins with 1 tropomyosin and 1 troponin complex, are aligned along the myocardial thin filament in a head-to-tail manner. A central RU in the *B* state may have 9 possible nearest neighbor RU-RU configurations (Fig. 8, manuscript):

(1) *BB* - both neighbors blocked;

(2) *BC* - left neighbor blocked and right neighbor closed;

(3) *CB* - left neighbor closed and right neighbor blocked;

(4) *BM* - left neighbor blocked and right neighbor open;

(5) *MB* - left neighbor open and right neighbor blocked;

(6) *CC* - both neighbors closed;

(7) *CM* - left neighbor closed and right neighbor open;

(8) *MC* - left neighbor open and right neighbor closed;

(9) *MM* - both neighbors open.

1. Blocked and closed transitions: k_{BC} and k_{CB}

In this subsection, we examine the effects of interactions between neighboring regulatory units on the blocked-to-closed (i.e., k_{BC} and k_{CB}) and closed-to-open (i.e., k_{CM_1} and k_{M_1C}) transitions (Fig. 2, manuscript). We assume that the activation energy needed for the *B* to *C* transition of a single RU depends on nearest neighbor RUs according to the formula:

$$E_{01}^{xy} = E_{01}^{\text{ref}} + E_{01}^{x} + E_{01}^{y}, \qquad x, y \in \{0,1,2\},$$

where E_{01}^{ref} is the activation energy associated with the transition from state 0 to state 1 under reference conditions in which both neighbors are in the C state; E_{01}^{x} and E_{01}^{y} represent additive contributions of either neighboring RU, having state *x* and state *y* respectively, to the activation energy for transition from 0 to 1. By Boltzmann statistics, the equilibrium constant of the blocked-to-closed transition, denoted by k_{BC}^{xy}, is given by

$$k_{BC}^{xy} = \exp\left\{-\frac{E_{01}^{xy}}{\kappa T}\right\}$$

in which κ is the Boltzmann constant and *T* is the absolute temperature. In fact, the term k_{BC}^{xy} can be interpreted as the probability that an attempt to make a transition from 0 to 1 will be successful when nearest neighbors of a RU are in the *x* and *y* states, respectively. This

means that the higher the activation energy E_{01}^{xy}, the smaller the probability of success. Now we let

$$k_{BC}^{\text{ref}} = \exp\left\{-\frac{E_{01}^{\text{ref}}}{\kappa T}\right\} =: k_{BC}^{CC} \text{ and } \gamma(xy) = \exp\left\{-\frac{E_{01}^{x} + E_{01}^{y}}{\kappa T}\right\}.$$

to define the cooperative coefficients for the blocked-to-closed transition as

$$u_1 = \exp\left\{\frac{E_{01}^{0}}{\kappa T}\right\} \text{ and } u_2 = \exp\left\{-\frac{E_{01}^{2}}{\kappa T}\right\}.$$

With the definitions of u_1 and u_2, $\gamma(xy)$ takes on the neighbor-dependent values,

$$\begin{aligned}
&\gamma(BB) = u_1^{-2}, \\
&\gamma(BC) = \gamma(CB) = u_1^{-1}, \\
&\gamma(BM) = \gamma(MB) = u_1^{-1}u_2, \\
&\gamma(CC) = 1, \\
&\gamma(CM) = \gamma(MC) = u_2, \\
&\gamma(MM) = u_2^2.
\end{aligned}$$

Thus, the neighbor-dependent equilibrium constant of the blocked-to-closed transition of one single RU is given by:

$$k_{BC}^{xy} = k_{BC}^{\text{ref}}\gamma(xy) \qquad x, y \in \{B, C, M\}.$$

By considering the whole thin filament, since there are 9 possible nearest neighbor conformations of two nearest neighboring RUs as listed above, the transition rate k_{BC} can be expressed as

$$k_{BC} = k_{BC}^{a} \sum_{x,y \in \{B,C,M\}} \lambda^x \lambda^y k_{BC}^{xy}$$

in which k_{BC}^{a} is an attempt frequency for the transition from *B* to *C*, λ^x is the probability that a neighboring site will be in state *x*, λ^y is the probability that a neighboring site will be in state

y, and hence the product $\lambda^x\lambda^y$ is the joint probability that the left neighbor will be *x* and the right one will be *y*. By computation,

$$\begin{aligned} k_{BC} &= k_{BC}^{a}k_{BC}^{\text{ref}}\left[(\lambda^B)^2u_1^{-2}+2\lambda^B\lambda^C u_1^{-1}+2\lambda^B\lambda^M u_1^{-1}u_2+(\lambda^C)^2+2\lambda^C\lambda^M u_2+(\lambda^M)^2u_2^2\right] \\ &= k_{BC}^{a}k_{BC}^{\text{ref}}\left(\lambda^B u_1^{-1}+\lambda^C+\lambda^M u_2\right)^2. \end{aligned}$$

We let $k_{BC}^{(1,1)} = k_{BC}^{a}k_{BC}^{\text{ref}}$, which is a reference k_{BC} coefficient for the condition where both neighbors are in the *C* state, then, since $\lambda^C = 1-\lambda^B-\lambda^M$, we obtain

$$k_{BC} := k_{BC}^{(u_1,u_2)} = k_{BC}^{(1,1)}\left[1-\lambda^B(1-u_1^{-1})+\lambda^M(u_2-1)\right]^2. \qquad \textbf{\textit{(Eq. 7)}}$$

In an analogous manner, the activation energy required for the closed-to-open transition of a single RU that depends on nearest neighbor RUs is of the form,

$$E_{10}^{xy} = E_{10}^{\text{ref}} + E_{10}^{x} + E_{10}^{y} \qquad\qquad x,y\in\{0,1,2\}.$$

According to Boltzmann statistics,

$$k_{CB}^{xy} = \exp\left\{-\frac{E_{10}^{xy}}{\kappa T}\right\} \text{ and } k_{CB}^{\text{ref}} = \exp\left\{-\frac{E_{10}^{\text{ref}}}{\kappa T}\right\} =: k_{CB}^{CC}.$$

Notice that $E_{01}^{0} = -E_{10}^{0}$ and $E_{01}^{2} = -E_{10}^{2}$. Hence the neighbor-dependent equilibrium constant of the closed-to-blocked transition of a single RU is of the form,

$$k_{CB}^{xy} = k_{CB}^{\text{ref}}\gamma(xy)^{-1} \qquad\qquad x,y\in\{B,C,M\}.$$

The same argument as above implies that the transition rate k_{CB} along the whole thin filament is given by,

$$k_{CB} := k_{CB}^{(u_1,u_2)} = k_{CB}^{(1,1)}\left[1+\lambda^B(u_1-1)-\lambda^M(1-u_2^{-1})\right]^2. \qquad \textbf{\textit{(Eq. 8)}}$$

where $k_{CB}^{(1,1)} = k_{CB}^{a} k_{CB}^{\text{ref}}$ is a reference k_{CB} coefficient for the condition where both neighbors are in the *C* state and k_{CB}^{a} is an attempt frequency for the transition from the closed-to-block state.

Note that, because the effects of Ca^{2+} on k_{BC} and k_{CB} are independent of the effects of neighbor interactions, $k_{BC}^{(1,1)}$ and $k_{CB}^{(1,1)}$ incorporate the Ca^{2+} effect as given by ***Eqs. 5*** and ***6***. As the more neighbors assume the *B* state, the more activation energy must be overcome for the transition from 0 to 1 to be successful. So, the neighboring RUs in the *B* state tend to increase the activation energy E_{01}^{xy}, i.e., $E_{01}^{0} \geq 0$ (Fig. 8, manuscript). Similarly, the neighboring RUs in the *M* state have tendency to reduce the activation energy E_{01}^{xy} and hence $E_{01}^{2} \leq 0$. If $E_{01}^{0} = E_{01}^{2} = 0$ then $u_1 = u_2 = 1$ and there is no additional contribution of the neighboring RU in the state *B* to the activation energy for the transition from 0 to 1. This means that there is no effect of near neighbor interactions. However, if either $E_{01}^{0} > 0$ or $E_{01}^{2} < 0$ then either $u_1 > 1$ or $u_2 > 1$, meaning that the system of interacting near neighbor RUs are more tightly coupled, and therefore more cooperative.

<u>2.</u> *<u>Closed and open transitions</u>:* k_{CM_1} and k_{M_1C}

We denote by E_{12}^{ref} the activation energy required for the transition from state 1 to state 2 under reference conditions where both neighbors are in the *C* state, E_{12}^{x} and E_{12}^{y} additive contributions of a neighboring RU in state *x* and a neighboring RU in state *y* to the activation energy for the transition from 1 to 2. Then the activation energy needed for the closed-to-open transition of a single RU that depends on nearest neighbor RUs takes the form

$$E_{12}^{xy} = E_{12}^{\text{ref}} + E_{12}^{x} + E_{12}^{y} \qquad x, y \in \{0,1,2\}.$$

The notion underlying this formulation is that RU-RU coupling imposes additional energy changes on the transitions that result from neighboring RUs occupying dissimilar states. More specifically, E_{12}^{0} increases the activation energy for the transition from 1 to 2 while E_{12}^{2} decreases this activation energy. Hence $E_{12}^{0} \geq 0 \geq E_{12}^{2}$. By Boltzmann statistics,

$$k_{CM_1}^{xy} = \exp\left\{-\frac{E_{12}^{xy}}{\kappa T}\right\}$$

This represents the probability that an attempt to make the transition from 1 to 2 will be successful with a left near neighbor of a RU in state *x* and the right neighbor in state *y*

$$k_{CM_1}^{\mathrm{ref}} = \exp\left\{-\frac{E_{12}^{\mathrm{ref}}}{\kappa T}\right\} =: k_{CM_1}^{CC} \text{ and } \mu(xy) = \exp\left\{-\frac{E_{12}^{x} + E_{12}^{y}}{\kappa T}\right\}.$$

By defining the cooperative coefficients as

$$z_1 = \exp\left\{\frac{E_{12}^{0}}{\kappa T}\right\} \text{ and } z_2 = \exp\left\{-\frac{E_{12}^{2}}{\kappa T}\right\}.$$

the neighbor-dependent equilibrium constant of the *C*-to-*M*1 transition is given by

$$k_{CM_1}^{xy} = k_{CM_1}^{\mathrm{ref}} \mu(xy) \qquad\qquad x, y \in \{B, C, M\}$$

with $\mu(xy)$ taking on the values,

$$\begin{aligned}
&\gamma(BB) = z_1^{-2},\\
&\gamma(BC) = \gamma(CB) = z_1^{-1},\\
&\gamma(BM) = \gamma(MB) = z_1^{-1} z_2,\\
&\gamma(CC) = 1,\\
&\gamma(CM) = \gamma(MC) = z_2,\\
&\gamma(MM) = z_2^{2}.
\end{aligned}$$

Using the same reasoning as with the *B*-to-*C* transition, along the whole thin filament we obtain,

$$k_{CM_1} := k_{CM_1}^{(z_1,z_2)} = k_{CM_1}^{(1,1)}\left[1-\lambda^B(1-z_1^{-1})+\lambda^M(z_2-1)\right]^2, \qquad \textbf{(Eq. 9)}$$

where $k_{CM_1}^{(1,1)} = k_{CM_1}^a k_{CM_1}^{\mathrm{ref}}$ and $k_{CM_1}^a$ is the attempt frequency of the transition from 1 to 2. In the similar notations and arguments, the activation energy required for the M_1-to-C transition of a single RU depending on nearest neighbor RUs has the form

$$E_{21}^{xy} = E_{21}^{\mathrm{ref}} + E_{21}^{x} + E_{21}^{y}.$$

By Boltzmann statistics,

$$k_{M_1C}^{xy} = \exp\left\{-\frac{E_{21}^{xy}}{\kappa T}\right\} \text{ and } k_{M_1C}^{\mathrm{ref}} = \exp\left\{-\frac{E_{21}^{\mathrm{ref}}}{\kappa T}\right\} =: k_{M_1C}^{CC}.$$

Since $E_{21}^0 = -E_{12}^0$ and $E_{21}^2 = -E_{12}^2$, the neighbor-dependent equilibrium constant of the M_1-to-C transition of a single RU is of the form

$$k_{M_1C}^{xy} = k_{M_1C}^{\mathrm{ref}}\mu(xy)^{-1} \qquad x,y \in \{B,C,M\}.$$

Therefore, by the same reasoning as in k_{CM_1}, along the whole thin filament we obtain:

$$k_{M_1C} := k_{M_1C}^{(z_1,z_2)} = k_{M_1C}^{(1,1)}\left[1+\lambda^B(z_1-1)-\lambda^M(1-z_2^{-1})\right]^2. \qquad \textbf{(Eq. 10)}$$

where $k_{M_1C}^{(1,1)} = k_{M_1C}^a k_{M_1C}^{\mathrm{ref}}$ and $k_{M_1C}^a$ is the attempt frequency of the transition from 2 to 1. Thus, the cooperative coefficients z_1 and z_2 measures the strength of neighboring RU-RU interactions on the transition rates k_{CM_1} *and* k_{M_1C}, respectively. If $z_1 = z_2 = 1$ there is no effect of neighboring RU-RU interactions on these transition rates. However, if $z_1 > 1$ or $z_2 > 1$, the system of interacting near neighbor RUs becomes more cooperative.

B. XB-XB interactions

We now consider that the force-generating state of an attached XB (i.e., *M2*) reduces the activation energy for the *C*-to-M_1 transition by increasing the rate coefficient k_{CM_1} and decreasing the reverse rate coefficient k_{M_1C} (Fig. 3, manuscript).

In this subsection, we can label the non-force-generating state as "0", which includes the *B* or *C* or M_1 states. We denote by $E_{CM_1}^{\text{ref}} = E_{CM_1}^{00}$ the activation energy required for the *C*-to-M_1 transition to occur under reference conditions in which both nearest neighboring XBs are in the non-force-generating state, and $E_{CM_1}^{xy}$ the activation energy needed for the *C*-to-M_1 transition to occur when both nearest neighboring XBs are in state *x* and state *y*, respectively, in which $x, y \in \{0, M_2\}$. Any XB, whether in the non-force-generating state or force-generating state, has 4 possible nearest neighbor configurations:

1. 00 - neither of neighbors are in the force-generating state;
2. $0M_2$ - the left neighbor is in the non-force-generating state and the right one is in the force-generating state;
3. M_20 - the left neighbor is in the force-generating state and the right one is in the non-force-generating state; and
4. M_2M_2 - both of neighbors are in the force-generating states.

In a manner similar to that described for RU-RU interactions, the transition rate k_{CM_1} can be estimated along the whole thin filament when the effects of neighboring XB attachment sites are taken into consideration as follows

$$k_{CM_1} = f^a_{CM_1}\left[(\lambda^B+\lambda^C+\lambda^{M_1})(\lambda^B+\lambda^C+\lambda^{M_1})\exp\left\{-\frac{E^{00}_{CM_1}}{\kappa T}\right\} + 2(\lambda^B+\lambda^C+\lambda^{M_1})\lambda^{M_2}\exp\left\{-\frac{E^{0M_2}_{CM_1}}{\kappa T}\right\} + \lambda^{M_2}\lambda^{M_2}\exp\left\{-\frac{E^{M_2M_2}_{CM_1}}{\kappa T}\right\}\right]$$

where $f^a_{CM_1}$ is an attempt frequency, $(\lambda^B+\lambda^C+\lambda^{M_1})(\lambda^B+\lambda^C+\lambda^{M_1})$ is the probability that both neighboring XBs will be in the non-force-generating state, $2(\lambda^B+\lambda^C+\lambda^{M_1})\lambda^{M_2}$ is the probability that one of neighboring XB will be in the force-generating state, and $\lambda^{M_2}\lambda^{M_2}$ is the probability that both of neighboring XBs will be the force-generating state. The above expression can be rewritten as,

$$k_{CM_1} = f^a_{CM_1}\exp\left\{-\frac{E^{00}_{CM_1}}{\kappa T}\right\}\left[\left(\lambda^B+\lambda^C+\lambda^{M_1}\right)\left(\lambda^B+\lambda^C+\lambda^{M_1}\right) + 2(\lambda^B+\lambda^C+\lambda^{M_1})\lambda^{M_2}\exp\left\{-\frac{E^{0M_2}_{CM_1}-E^{00}_{CM_1}}{\kappa T}\right\} + \lambda^{M_2}\lambda^{M_2}\exp\left\{-\frac{E^{M_2M_2}_{CM_1}-E^{00}_{CM_1}}{\kappa T}\right\}\right].$$

We assume that the reduction in activation energy is proportional to the force generated by the XB at the neighboring site. So

$$E^{0M_2}_{CM_1} - E^{00}_{CM_1} = -VF_{M_2}$$
$$E^{M_2M_2}_{CM_1} - E^{00}_{CM_1} = -2VF_{M_2}$$

where V is the constant of proportionality and F_{M_2} is the force associated with the force-generating attached XBs at neighboring sites. Therefore, if we let

$f^0_{CM_1} = f^a_{CM_1}\exp\{-\frac{E^{00}_{CM_1}}{\kappa T}\}$ that refers to the condition when no neighbors are in the force-generating state, then

$$k_{CM_1} = f^0_{CM_1}\left[(\lambda^B+\lambda^C+\lambda^{M_1})^2 + 2(\lambda^B+\lambda^C+\lambda^{M_1})\lambda^{M_2}\exp\left\{\frac{VF_{M_2}}{\kappa T}\right\} + (\lambda^{M_2})^2\exp\left\{\frac{2VF_{M_2}}{\kappa T}\right\}\right]$$
$$= f^0_{CM_1}\left[\lambda^B+\lambda^C+\lambda^{M_1}+\lambda^{M_2}\exp\left\{\frac{VF_{M_2}}{\kappa T}\right\}\right]^2$$

which follows that

$$k_{CM_1} = f^0_{CM_1}\left[1+\lambda^{M_2}\left(\exp\left\{\frac{VF_{M_2}}{\kappa T}\right\}-1\right)\right]^2.$$

According to elastic XB theory and under the isometric conditions (Razumova et al., 1999; Campbell et al., 2001), $F_{M_2} = \xi x_0$ where ξ is the stiffness of a single XB and x_0 is the average distortion among XBs of M_2 state. This distortion may be visualized as being induced during a strongly bound, post-isomerization state where the XB head has rotated to put distortion equal to x_0. If we let $v-1=\frac{\xi V x_0}{\kappa T}$, then v represents a cooperative parameter that measures the strength of neighboring XB-XB interactions on the transition rates k_{CM_1} and k_{M_1C}. Therefore,

$$k_{CM_1} := k^v_{CM_1} = f^0_{CM_1}\left[1+\lambda^{M_2}(e^{v-1}-1)\right]^2. \qquad \textbf{\textit{(Eq. 11)}}$$

$$k_{M_1C} := k^v_{M_1C} = f^0_{M_1C}\left[1+\lambda^{M_2}(e^{-v+1}-1)\right]^2. \qquad \textbf{\textit{(Eq. 12)}}$$

If v = 1 there is no cooperative interaction between nearest neighbor XBs. If v > 1 then XB-XB interactions are more tightly coupled, and hence, more cooperative.

C. XB-RU interactions

We can now consider that the *B*-to-*C* transition of RUs is favored by the force-generating state of XBs at neighboring actin-myosin attachment sites (Fig. 4, manuscript). We allow that

a force-generating XB at a neighboring site reduces the activation energy needed for the B-to-C transition. We denote by $E_{BC}^{\text{ref}} = E_{BC}^{00}$ the activation energy required for the B-to-C transition under reference conditions where both nearest neighboring XBs are in the non-force-generating state, and E_{BC}^{xy} the activation energy needed for the B-to-C transition as both nearest neighboring XBs are, respectively, in state x and state y where $x, y \in \{0, M_2\}$. By the same manner as described for RU-RU interactions, the transition rate k_{BC} can be computed along the whole thin filament when effects of neighboring XBs at attachment sites are considered as below,

$$k_{BC} = f_{BC}^{a} \exp\left\{-\frac{E_{BC}^{00}}{\kappa T}\right\}\Big[\left(\lambda^{B} + \lambda^{C} + \lambda^{M_1}\right)\left(\lambda^{B} + \lambda^{C} + \lambda^{M_1}\right)$$
$$+ 2(\lambda^{B} + \lambda^{C} + \lambda^{M_1})\lambda^{M_2} \exp\left\{-\frac{E_{BC}^{0M_2} - E_{BC}^{00}}{\kappa T}\right\} + \lambda^{M_2}\lambda^{M_2} \exp\left\{-\frac{E_{BC}^{M_2M_2} - E_{BC}^{00}}{\kappa T}\right\}\Big].$$

We assume that the reduction in activation energy is due to mechanical mechanisms, which depends on the amount of force in the XBs at neighboring sites. Therefore, we can write,

$$E_{BC}^{0M_2} - E_{BC}^{00} = -WF_{M_2}$$
$$E_{BC}^{M_2M_2} - E_{BC}^{00} = -2WF_{M_2}$$

where W is the constant of proportionality and F_{M_2} is the force associated with the force-generating attached XBs at neighboring sites. By letting $f_{BC}^{0} = f_{BC}^{a} \exp\{-\frac{B_{BC}^{\text{ref}}}{\kappa T}\}$ this represents a reference value of the transition rate k_{BC} when there is no force-generating XB at neighboring sites. We then proceed as we did previously with the transition rate k_{CM_1} to obtain

$$k_{BC} = f_{BC}^{0}\left[1 + \lambda^{M_2}\left(\exp\left\{\frac{WF_{M_2}}{\kappa T}\right\} - 1\right)\right]^2.$$

Since $F_{M_2} = \xi x_0$, $\frac{WF_{M_2}}{\kappa T} = \frac{\xi W x_0}{\kappa T}$, we let $w - 1 = \frac{\xi W x_0}{\kappa T}$, in which *w* represents a cooperative parameter that measures the strength of neighboring XB-RU interactions on the transition rates k_{BC} and k_{CB}. So

$$k_{BC} := k_{BC}^{w} = f_{BC}^{0}\left[1 + \lambda^{M_2}(e^{w-1} - 1)\right]^2. \qquad \textbf{\textit{(Eq. 13)}}$$

$$k_{CB} := k_{CB}^{w} = f_{CB}^{0}\left[1 + \lambda^{M_2}(e^{-w+1} - 1)\right]^2. \qquad \textbf{\textit{(Eq. 14)}}$$

If *w* = 1 there is no cooperative XB-RU interaction. If w > 1, then the XB-RU interactions are more tightly coupled, and hence, more cooperative. It should be noted that due to the independence between Ca^{2+} effect and neighboring interaction effect, f_{BC}^{0} and f_{CB}^{0} incorporate the Ca^{2+} effect as given by ***Eqs. 5*** and ***6***, respectively.

D. Ensemble effects of RU-RU, XB-XB, and XB-RU near neighbor interactions

Finally, we can integrate the three nearest neighbor RU-RU, XB-XB, and XB-RU interactions into our thin filament model. We assume that the *B*-to-*C* transition k_{BC} of an RU can be simultaneously impacted by the configurations of its two neighboring RUs and configurations of cross-bridges at two neighboring actin-myosin attachment sites. This means that the *B*-to-*C* transition can be rewritten as

$$k_{BC} = f_{BC}^{0}\left\{\alpha\left[1 - \lambda^{B}(1 - u_1^{-1}) + \lambda^{M}(u_2 - 1)\right]^2 + (1-\alpha)\left[1 + \lambda^{M_2}(e^{w-1} - 1)\right]^2\right\}, \qquad \textbf{\textit{(Eq. 15)}}$$

in which $0 \le \alpha \le 1$ is a measure of the extent to which k_{BC} is affected by RU-RU interactions and $1-\alpha$ measures the extent of the effect of XB-RU interactions on k_{BC}. For example, when $\alpha = 0$, k_{BC} is unaffected by RU-RU interactions but is only affected by XB-RU interactions. In a similar manner, the reverse *C*-to-*B* transition can be written as

$$k_{CB} = f_{CB}^{0}\left\{\bar{\alpha}\left[1+\lambda^{B}(u_1-1)-\lambda^{M}(1-u_2^{-1})\right]^2+(1-\bar{\alpha})\left[1+\lambda^{M_2}(e^{-w+1}-1)\right]^2\right\}, \quad \textbf{\textit{(Eq. 16)}}$$

where $0 \le \bar{\alpha} \le 1$ and $1-\bar{\alpha}$ represent the measures of the extent to which RU-RU and XB-RU interactions have effects on k_{CB}, respectively. For instance, when $\bar{\alpha}=1$, only RU-RU interactions affect the transition rate k_{CB}. In a similar fashion, we also assume that the effects of the RU-RU interaction and XB-XB interaction on the C-to-M_1 transition and the M_1-to-C transition can be taken into account as follows:

$$k_{CM_1} = f_{CM_1}^{0}\left\{\beta\left[1-\lambda^{B}(1-z_1^{-1})+\lambda^{M}(z_2-1)\right]^2+(1-\beta)\left[1+\lambda^{M_2}(e^{v-1}-1)\right]^2\right\}, \quad \textbf{\textit{(Eq. 17)}}$$

$$k_{M_1C} = f_{M_1C}^{0}\left\{\bar{\beta}\left[1+\lambda^{B}(z_1-1)-\lambda^{M}(1-z_2^{-1})\right]^2+(1-\bar{\beta})\left[1+\lambda^{M_2}(e^{-v+1}-1)\right]^2\right\}. \quad \textbf{\textit{(Eq. 18)}}$$

where β and $1-\beta$ represent the extent to which RU-RU and XB-XB interactions effect k_{CM_1} and where $\bar{\beta}$ and $1-\bar{\beta}$ represent the extent to which RU-RU and XB-XB interactions effect k_{M_1C}. For convenience, we designate the parameters $(\alpha,\bar{\alpha},\beta,\bar{\beta})$ as nearest neighbor interaction factors. We can use the nearest neighbor interaction factors to quantify the relative contributions of RU-RU, XB-XB, and XB-RU interactions on the overall dynamics of the model via transition rates k_{BC}, k_{CB}, k_{CM_1}, and k_{M_1C}. For example, we can formulate one single parameter α to link the effect of RU-RU and XB-RU interactions together on k_{BC} in an antagonistic way. If $\alpha=0.9$ then the relative contribution of RU-RU interactions on k_{BC} is 90%, and due to our assumption, XB-RU interactions only contribute 10% to k_{BC}. This means that RU-RU interactions dominate the tandem effect. Conversely, if $\alpha=0.1$ then XB-RU interactions play a more dominant role. If $\alpha=0.5$ then both RU-RU and XB-RU equally

contribute to the tandem effect on k_{BC}. The use of the factors $(\alpha,\bar{\alpha},\beta,\bar{\beta})$ as balancing coefficients between nearest neighbor interactions simplifies the model, making it analytically tractable when fitting it to mechanical data in mouse and porcine, while capturing features of the competitive dynamics between cooperative mechanisms. This allows for mathematical simplicity and clearer interpretation of how changes in one type of near neighbor interaction influence the overall behavior of the system.

III. Reliability of model fitting

In this section, we evaluated the reliability of our model (***Eqs.*** **2, 3** and **4** with 20 parameters) when it was fitted to *k*tr vs. pCa data in both murine and porcine ventricular myocardium using model resolution matrices (Table S5). We examined the model resolution matrices generated by using parameter sets 1, 2, and 8 to assess the degree of parameter independence and the robustness of the parameter estimates. For both murine and porcine data, the model resolution matrices corresponding to Parameter Set 1 indicate that the parameters are generally well-solved (Fig S4, left panels). This is evidenced by the high values (i.e., close to 1) along the diagonal signifying that these numerical factors are independently estimated with minimal interdependence. The off-diagonal elements are predominantly low (i.e., close to 0), highlighting that there is minimal correlation between the fitted parameters. For the mouse data using Parameter Sets 2 and 8, we observed resolution matrices with well-resolved diagonal elements and low off-diagonal values, confirming the robustness and reliability of the parameter estimates (Figure S4, upper middle and right panels). This is especially apparent when examining the numerical factors 11 to 14 which designate the cooperative coefficients u_1, u_2, z_1, and z_2 for Parameter Sets 2 and 8 and show high values (yellow colors, that is, close to 1). These matrices imply that the cooperative

coefficients u_1, u_2, z_1, and z_2 play a significant role when our model is used to fit the in vitro contractility data from murine myocardium. For the porcine data, a similar trend is observed regarding the resolution matrices for parameter sets 2 and 8 (Fig S4, lower middle and right). In these matrices we observed strong diagonal elements, particularly for parameters 11-14 (corresponding to u_1, u_2, z_1, z_2), suggesting that these cooperative coefficients are critical and well-resolved. The low off-diagonal values further support the independence of the parameter estimates, indicating that the model reliably captures the system dynamics in the porcine myocardium. Overall, the resolution matrices modeling murine and porcine in vitro contractility data suggest that the fitted parameters are reliable and independently estimated. This underscores the robustness of our model in accurately representing the $k$tr vs. pCa relationship in ventricular myocardium in different species using our model parameter sets. Furthermore, these matrices demonstrate the indispensability of RU-RU cooperative coefficients u_1, u_2, z_1, z_2 incorporated within our model.

References

Campbell, K.B., M.V. Razumova, R.D. Kirkpatrick, and B.K. Slinker. 2001. Myofilament kinetics in isometric twitch dynamics. *Ann. Biomed. Eng.* 29:384-405.

Chen, P.P., J.R. Patel, I.N. Rybakova, J.W. Walker, and R.L. Moss. 2010. Protein kinase A-induced myofilament desensitization to Ca^{2+} as a result of phosphorylation of cardiac myosin-binding protein C. *J. Gen. Physiol.* 136:615-627.

Fabiato, A. 1988. Computer programs for calculating total from specified free or free from specified total ionic concentrations in aqueous solutions containing multiple metals and ligands. *Methods Enzymol.* 157:378-417.

Giles, J., J.R. Patel, A. Miller, E. Iverson, D. Fitzsimons, and R.L. Moss. 2019. Recovery of left ventricular function following in vivo reexpression of cardiac myosin binding protein C. *J. Gen. Physiol.* 151:77-89.

Godt, R.E., and B.D. Lindley. 1982. Influence of temperature upon contractile activation and isometric force production in mechanically skinned muscle fibers of the frog. *J. Gen. Physiol.* 80:279-297.

Patel, J.R., G.P. Barton, R.K. Braun, K.N. Goss, K. Haraldsdottir, A. Hopp, G. Diffee, T.A. Hacker, R.L. Moss, and M.W. Eldridge. 2017. Altered right ventricular mechanical properties are afterload dependent in a rodent model of bronchopulmonary dysplasia. Front. Physiol. 8:840.

Razumova, M.V., A.E. Bukatina, and K.B. Campbell. 1999. Stiffness-distortion sarcomere model for muscle stimulation. *J. Appl. Physiol.* 87:1861-1876.
